# Impacts of Ser/Thr Protein Kinase Stk1 on the Proteome, Twitching Motility, and Competitive Advantage in *Pseudomonas aeruginosa*

**DOI:** 10.3389/fmicb.2021.738690

**Published:** 2021-09-22

**Authors:** Xuan Zhu, Chao Feng, Lantian Zhou, Zhenzhen Li, Yue Zhang, Jianyi Pan

**Affiliations:** Zhejiang Provincial Key Laboratory of Silkworm Bioreactor and Biomedicine, College of Life Sciences and Medicine, Zhejiang Sci-Tech University, Hangzhou, China

**Keywords:** Ser/Thr protein kinase, Stk1, proteome, type IV pilus motility, T6SS-H1, *Pseudomonas aeruginosa*

## Abstract

*Pseudomonas aeruginosa* is a ubiquitous gram-negative bacterium in the environment and a leading cause of nosocomial infections worldwide. Therefore, it is listed by the WHO as a human pathogen that urgently needs the development of new antibacterial drugs. Recent findings have demonstrated that eukaryote-type Ser/Thr protein kinases play a vital role in regulating various bacterial physiological processes by catalyzing protein phosphorylation. Stk1 has proven to be a Ser/Thr protein kinase in *P. aeruginosa*. However, the regulatory roles of Stk1 have not yet been revealed. Thus, we constructed a *stk1* knockout mutant (∆*stk1*) from the *P. aeruginosa* PAO1 strain and employed a Tandem Mass Tag (TMT) labeling-based quantitative proteomic strategy to characterize proteome-wide changes in response to the *stk1* knockout. In total, 620 differentially expressed proteins, among which 288 proteins were upregulated and 332 proteins were downregulated, were identified in ∆*stk1* compared with *P. aeruginosa* PAO1. A detailed bioinformatics analysis of these differentially expressed proteins was performed, including GO annotation, protein domain profile, Kyoto Encyclopedia of Genes and Genomes (KEGG) pathway analysis, subcellular localization and enrichment analysis. Notably, the downregulation of type IV pilus-related proteins and upregulation of T6SS-H1-related proteins were found in the ∆*stk1* strain, and the results were corroborated by quantitative PCR at the mRNA level. Further experiments confirmed that the loss of *stk1* weakens bacterial twitching motility and promotes a growth competition advantage, which are, respectively, mediated by type IV pilus-related proteins and T6SS-H1-related proteins. These findings contribute to a better understanding of the physiological role of Stk1, and proteomic data will help further investigations of the roles and mechanisms of Stk1 in *P. aeruginosa*, although the detailed regulation and mechanism of Stk1 still need to be revealed.

## Introduction

*Pseudomonas aeruginosa* is a ubiquitous gram-negative bacterium in the environment and is one of the top three pathogens causing opportunistic human infections. This bacterium has become an important pathogen not only due to its virulence but also because of its strong resistance to antibiotics and disinfectants (Stover et al., 2000). *Pseudomonas aeruginosa* has a strong ability to infect patients with low immune function, cystic fibrosis (*CF*), burns, open fractures, or implanted medical devices (such as catheters), accounting for 10–20% of nosocomial infections, and it is listed by the WHO as one of the human pathogens that urgently needs the development of new antimicrobial drugs (Lyczak et al., 2000). As we all know, the severity and the type of infection is highly dependent upon the degree of expression of virulence factors that can be epigenetically modulated (Serra et al., 2015; Newman et al., 2017; Azam and Khan, 2019). Bacterial virulence factors include toxins secreted into the environment, which can be large molecules such as elastase and phospholipase or small molecules such as pyocyanin, rhamnolipid, and cyanide. In addition, by using adhesion factors such as type IV pilus to adhere to host cells, bacteria can mediate biofilm formation and virulence factor production and secretion (O’toole and Kolter, 1998; Hancock and Speert, 2000; Overhage et al., 2008). An important reason why *P. aeruginosa* successfully infects and causes persistent infection is its strong ability to form biofilms, which makes it extremely resistant to antibiotics, leading to difficulties in clinical treatment (Venturi, 2006). Therefore, it is urgent to develop new antibiotic drugs on the basis of comprehensively revealing the resistance mechanism of *P. aeruginosa*.

Recent studies have shown that protein phosphorylation modification is widespread in bacteria and plays an important role in regulating various physiological processes, including several key steps in the process of pathogen infection, such as adhesion to host cells, regulating pathogenic function and virulence, disrupting signaling and damaging the host defense mechanism, and drug resistance (Rahme et al., 2000; Braun et al., 2008; Mijakovic and Macek, 2012; Little et al., 2018; Hoffman et al., 2020). A typical phosphorylation enzyme is Ser/Thr protein kinase. In recent years, eukaryotic-like Ser/Thr protein kinases and Tyr protein kinases have been gradually discovered in prokaryotes. These kinases and other types of protein kinases form a complex phosphorylation network in prokaryotes (Jers et al., 2008). Although Ser/Thr protein kinases are not DNA-binding proteins, they can mediate the phosphorylation of substrate proteins to regulate gene expression, affect the cellular localization, metabolism and many other cellular functions (Bonne Køhler et al., 2020). For example, the Ser/Thr protein kinase PrkC in *Bacillus anthracis* can mediate the expression of the metabolic protein Eno, which acts as an intrinsic memory controller and affects the germination process, thereby helping *B. anthracis* survive the nutritional shift and promoting the role of glycolytic enzymes in the carbohydrate metabolic process during germination (Virmani et al., 2019). Similar mechanisms also occur in the bacterial regulation of the secretion and release of virulence factors. For example, the membrane-localized protein kinase Stk1 in *Streptococcus agalactiae* regulates the activity of pyrophosphatase and other cellular functions, which leads to decreased bacterial virulence (Rajagopal et al., 2003). PknB is a Ser/Thr protein kinase in *Staphylococcus aureus*, and the inactivation of PknB by mutating the kinase domain of the enzyme results in reduced bacterial virulence (Débarbouillé et al., 2009). In bacteria, Ser/Thr protein kinases may cooperate with other types of kinases, resulting in double or even multiple modifications of transcriptional regulators, thus playing an important role in cell wall synthesis, bacterial division, spore formation, bacterial metabolism, and immune modification (Rajagopal et al., 2003; Débarbouillé et al., 2009; Virmani et al., 2019).

Genomic analysis showed that *P. aeruginosa* may contain at least three Ser/Thr protein kinases, PpkA, Stk1, and PA1782 (Sana et al., 2012). Stk1 can phosphorylate histone H1, a common substrate used to verify Ser/Thr protein kinase activity, which confirms that Stk1 is a Ser/Thr protein kinase (Mukhopadhyay et al., 1999). Studies have shown that the Ser/Thr protein kinase PpkA is involved in the regulation of virulence factors and biofilm formation in *P. aeruginosa* (Goldová et al., 2011). However, the regulatory roles of Stk1 in the virulence and drug resistance in *P. aeruginosa* have not yet been revealed. Therefore, elucidating the regulatory mechanism of Stk1 will help us to understand the mechanisms of growth, development, pathogenicity, and drug resistance in *P. aeruginosa*. In this study, we constructed an *stk1* knockout strain (named Δ*stk1*) from the wild-type *P. aeruginosa* strain PAO1. A quantitative proteomics strategy based on TMT labeling and liquid phase tandem mass spectrometry was used to study the protein expression changes of the Δ*stk1* strain, and some biological effects mediated by differentially expressed proteins were analyzed.

## Materials and Methods

### Bacterial Strains, Plasmids, and Culture Conditions

The *P. aeruginosa* PAO1, *Escherichia coli* SM10-λpir and suicide plasmid pRE112 were maintained in our laboratory. The *P. aeruginosa* PAO1 was used as the wild-type strain in this study. The *P. aeruginosa* PAO1, Δ*stk1* mutant strain, and *E. coli* were all cultured at 37°C in Luria-Bertani (LB) medium with shaking at 200rpm or on LB agar plates [1.5% (w/v) agar in LB]. In addition, cetrimide agar medium was used as a selective medium for isolation of *P. aeruginosa* from *E. coli* strains. When required, the antibiotic chloromycetin was supplemented at a concentration of 25μg/ml in LB medium or 300μg/ml in cetrimide agar medium.

### Construction of Δ*stk1* Strain

For the gene knockout, a *sacB*-based strategy was employed as described in our previous studies (Liang et al., 2014). We constructed the *stk1*-deletion mutant strain (Δ*stk1*) from the *P. aeruginosa* PAO1 strain using the oligonucleotide primers shown in Table 1. PCR was performed to amplify an upstream fragment (453bp) and a downstream fragment (349bp) of *stk1* by using primers Δ*stk1*-F1/R1 (containing restriction sites for *Xba* I) and Δ*stk1*-F2/R2 (containing restriction sites for *Sac* I). The two PCR products were amplified by fusion PCR, and the products were then digested and cloned into the *Xba* I/*Sac* I-digested plasmid pRE112. The resulting recombinant plasmid, pRE112-Δ*stk1*, was transformed into *P. aeruginosa* PAO1 and screened in cetrimide agar medium with chloramphenicol resistance. The resulting colonies were further screened for loss of sucrose sensitivity (12% sucrose), which typically indicates a double crossover event and thus the occurrence of gene replacement. The Δ*stk1* mutant was further confirmed by using PCR primers Δ*stk1*-F3/R3, Δ*stk1*-F4/R4, and *sacB*-F/R (Table 1).

**Table 1:** Oligonucleotide primers used for construction of the *stk1*-deletion mutant and qPCR.

Primers	Sequence (5'–3')
For construction of the *stk1*-deletion mutant	
Δ*stk1*-F1	CTAGTCTAGAGGGCGAGGAACTCACCCTC
Δ*stk1*-R1	AGAGGTCCTGCAAACGTCAGGTTGTCCCGT
Δ*stk1*-F2	ACGGGACAACCTGACGTTTGCAGGACCTCT
Δ*stk1*-R2	TACGAGCTCCGTTTCTACGCCTCGG
Δ*stk1*-F3	ATGAACGAACCGCTGTCGTCGCTG
Δ*stk1*-R3	TCAACGGGCAAGAACGCCGGCC
Δ*stk1*-F4	CGGATGATCGTCACCAGCCT
Δ*stk1*-R4	ATCGCGCACGTGGAAATACC
pRE112-*sacB*-F	TACCTGCCGTTCACTATTATTTAGTG
pRE112-*sacB*-R	GGCGTGTAATATGGGAAATGC
For qPCR	
16S rRNA-F	ATACGTTCCCGGGCCTTGTA
16S rRNA-R	GTTCCCCTACGGCTACCTTG
*fimU*-F	TCACCCTGATCGAGTTGCTG
*fimU*-R	CGTACTGCAGCATCGCATTG
*pilW*-F	GACGCTTCGCCATGATGTTC
*pilW*-R	GGTTGAGCCGGCTTTGAATG
*amrZ*-F	TGAGCAGATCGCAGAAGTCG
*amrZ*-R	AGGCGAACACCGAGATTGTC
*pilV*-F	CTTCTTCAAGGCCAAGGGGT
*pilV*-R	GTAGTCGCTCTTCAGCAGGT
*vgrGb1*-F	CAAGAGCTTCACGCTCAACG
*vgrGb1*-R	GATGGTGATGTTCTTGCCGC
*tssB1*-F	TGCAGATCGAGTACGACGTG
*tssB1*-R	TCGATCTCCAGGAACTTGCG

### Preparation of Proteins, Digestion, and TMT Labeling

The preparation of proteins and digestion were carried out as described in our previous work (Ye et al., 2021). The *P. aeruginosa* wild-type strain PAO1 and Δ*stk1* mutant strain were both cultured in LB medium overnight. Cells were centrifuged at 6000×*g* for 20min at 4°C and washed with PBS three times. The resulting bacterial cell pellets were frozen and lyophilized and then stored at −80°C. The frozen bacterial cells were resuspended in lysis buffer [8M urea, 50mM Tris, 10mM EDTA, 10mM DTT, and 1% (v/v) protease inhibitor cocktail set III (Calbiochem), pH 8.0], mixed by pipetting and placed on ice for sonication. The resulting samples were centrifuged at 10,000×*g* for 15min at 4°C to remove unbroken cells and debris. Then, the supernatant was collected, and the protein in the supernatant was quantified by using a 2-D Quant kit (GE Healthcare). The resulting proteins were precipitated with ice-cold acetone overnight at −20°C, and the resulting precipitate was washed three times with ice-cold acetone. The pellets were air dried while the tubes were kept upside down. The air-dried precipitate was resuspended in 100mM NH_4_HCO_3_ and digested with trypsin (Promega) at an enzyme-to-substrate ratio of 1:50 for 14h at 37°C. Trypsin hydrolytic peptides were reduced with 5mM DTT at 56°C for 1h, followed by alkylation with 20mM iodoacetamide at room temperature in the dark for 30min. The reaction was terminated by incubation with 30mM cysteine at room temperature for 30min. To ensure complete digestion, trypsin was added at an enzyme to substrate ratio of 1:100, and the mixture was incubated for another 4h. Then, the resulting peptides were desalted by using a Strata X C18 SPE column (Phenomenex), followed by vacuum drying. The dried peptides were dissolved in 0.5M triethylammonium bicarbonate (TEAB) and labeled by incubation with Tandem Mass Tag (TMT) reagent (Pierce, United States) for 2h.

### Nano LC–MS/MS Analysis

Nano LC–MS/MS analysis were performed as described in our previous work (Ye et al., 2021). The TMT-labeling peptides were dissolved in solvent A (5mM NH_4_OH) and fractionated by high-pH reverse-phase HPLC coupled with an XBridge Shield C_18_ RP column (4.6mm i.d., 250mm length; Waters, United States) with a gradient of 5–80% solvent B (5mM NH_4_OH in 80% acetonitrile). The peptides were fractionated into 60 fractions in 90min and were combined into 12 fractions.

Each peptide fraction was analyzed by nano LC–MS/MS using an Ultimate 3,000 RSLCnano system (Thermo Scientific) coupled to a Q Exactive HF-X hybrid quadrupole-Orbitrap mass spectrometer (Thermo Scientific). The mass spectrometric analysis was performed in a data-dependent mode with an automatic switch between a full MS scan and an MS/MS scan. Peptides were detected in MS at a resolution of 70,000 with a scan range of 350–1800m/z and with automatic gain control (AGC) of 5e4. Peptides were selected for MS/MS using 26% normalized collision energy (NCE). The MS/MS scan was set as 110–1800m/z at a resolution of 30,000, and AGC was set as 1e6. The nano LC–MS/MS analysis was performed by Micrometer Biotech Company (Hangzhou, China).

### Database Searching and Protein Identification

All of the raw data files obtained from the HPLC–MS/MS analysis were processed using MaxQuant software against the protein database of *P. aeruginosa* from the Pseudomonas Genome DB.^1^ Trypsin/P was specified as the cleavage enzyme, and two missing cleavages and five charges were allowed. The oxidation of Met and acetyl (Protein *N*-term) was selected as the variable modification, and the carbamidomethylation of Cys was specified as a fixed modification. The mass error was set to 6ppm for precursor ions, and a fragment ion mass tolerance of 0.02Da was used. Otherwise, the default settings were used.

Proteins were annotated with GO terms from the Gene Ontology Consortium. Mass spectrometric analysis detects the changes of different peptide proteins and analyzes the sorted fold changes. Differentially expressed proteins were identified using a 1.3-fold change, and the data from triplicate experiments used the *t*-test to analyze statistical significance, where *p*<0.05 was considered significantly downregulated. Proteins that contained similar peptides and could not be distinguished based on mass spectrometry/mass spectrometry were grouped according to the principle of simplicity. The false discovery rate (FDR) of proteins and peptides was set to 1%. The minimum peptide length was 7. The critical value of the peptide was set to 40.

### Bioinformatics Analysis

Bioinformatics analysis of the identified proteins by using the software was described in detail previously (Pan et al., 2014). The gene ontology (GO) annotation proteome was derived from the UniProt-GOA database,^2^ and the proteins were classified based on three categories: biological process, cellular compartment, and molecular function. The Kyoto Encyclopedia of Genes and Genomes (KEGG) pathway was annotated by using the KEGG online tool KEGG Automatic Annotation Server (KAAS) to obtain the protein’s KEGG database description. The annotation results were mapped to the KEGG pathway database by using the KEGG online tool KEGG mapper, provided by Kanehisa Laboratories.^3^ The identified protein domain functional descriptions were annotated by InterProScan,^4^ a sequence analysis application, based on the protein sequence alignment method, and the InterPro domain database was used. Then, we used Wolfpsort^5^ to predict the subcellular localization. Wolfpsort uses an updated version of PSORT/PSORT II for the prediction of eukaryotic sequences.

For the members of the resulting protein clusters, the Fisher’s exact test was employed to test GO/KEGG pathway/InterPro domain enrichment analysis. Two-tailed Fisher’s exact test was used to test enriched pathways. InterPro was used to determine enriched domains.

For further category-based hierarchical clustering, we first sorted out all the categories and their value of *p* obtained after enrichment and then identified the categories that were enriched in at least one cluster with a value of *p*<0.05. The filtered value of *p* matrix was transformed by the function *x*=−log(*p* value), and the *x* value of each category was *z*-transformed. These *z* scores were then clustered by one-way hierarchical clustering (Euclidean distance, average linkage clustering) using Genesis. The cluster membership was visualized by a heat map using the heatmap.2 function in the ggplot2 R package.

### Twitching Motility Assay

The methods used for the study of motility were adopted from a previous study (Wu et al., 2011). Briefly, the twitching experiments were performed by using a toothpick to place the cell culture at an OD_600_ of 0.4 in the center of a plate with a basal surface of LB-1.0% Bacto agar, and the culture was incubated for 24h at 37°C. Cultures were stained with 0.5% crystal violet, and the diameter of the twitching zone was measured. The experiment was performed in triplicate.

### Growth Competition Assay

The methods used for the study of the competition were adopted from a previous study (Lin et al., 2015). The *P. aeruginosa* PAO1, Δ*stk1*, and *E. coli* K12 strains were cultured overnight, transferred to 100ml LB medium at a ratio of 1:100, cultured to an OD600 of 0.8 at 200rpm and 37°C, collected in 1ml culture and washed with PBS twice before mixing at a ratio of 1:1 in LB medium for a competition assay. The growth of PAO1 and Δ*stk1* in liquid LB medium alone was used as the noncompetition control. After incubating for 6h at 37°C and 200rpm, the cultures were spread on selective cetrimide agar plates at serial dilutions. The CFU values were determined by bacterial colony counting. At least three biological replicates were analyzed.

### RNA Extraction and Real-Time Quantitative PCR

The regulation of gene expression of several differentially expressed proteins in the Δ*stk1* strain compared with the wild-type strain PAO1 was analyzed by qPCR. The total RNA from bacterial cells was reverse-transcribed to cDNA using Hifair II 1st Strand cDNA Synthesis SuperMix for qPCR (gDNA digester plus). qPCR was performed in triplicate using Power SYBR® Green PCR Master Mix (Applied Biosystem) according to the manufacturer’s protocols. The oligonucleotide primers for qPCR are listed in Table 1. Thermal amplification was performed as follows: initial denaturation at 95°C for 1min, followed by 40cycles of 10s at 95°C and 30s at 60°C, and then a single fluorescence measurement. The relative gene expression was obtained using 16S rRNA as the control with an mRNA/16S rRNA of one in the wild-type strain. The gene expression data obtained from qPCR were evaluated using an ABI 7500 real-time PCR system.

### Statistical Analysis

The Student’s *t*-test was used to determine the statistical significance between pairs of experimental groups. Differences were considered significant when *p*<0.05. The two-tailed Fisher’s exact test was employed to test the GO/KEGG-pathway/InterPro enrichment analysis of protein domain and pathway. Correction for multiple hypothesis testing was carried out using standard false discovery rate control methods. The value of *p*<0.05 were considered statistically significant.

## Results and Discussion

### Construction of the Δ*stk1* Mutant Strain

**Figure 1:**
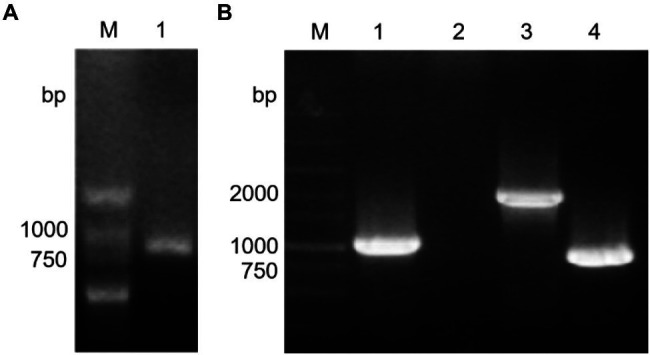
The construction of the ∆*stk1* mutant strain. **(A)** M: DNA marker; 1: the fragment containing the upstream and downstream homology arms of *stk1* was amplified by fusion PCR with primers ∆*stk1*-F1 and ∆*stk1*-R2 used to construct the recombinant plasmid pRE112-∆*stk1*. **(B)** M: DNA marker; 1 and 2: the DNA fragments of *stk1* were amplified by PCR with the primers ∆*stk1*-F3 and ∆*stk1*-R3 using the wild-type strain (lane 1) and ∆*stk1* strain (lane 2) chromosomal DNA as templates; 3 and 4: the long DNA fragments containing *stk1* were amplified by PCR with the primers ∆*stk1*-F4 and ∆*stk1*-R4 using the wild-type strain (lane 3) and ∆*stk1* strain (lane 4) chromosomal DNA as templates.

The Stk1 protein can phosphorylate histone H1, which is a substrate commonly used to verify Ser/Thr protein kinase activity (Mukhopadhyay et al., 1999), proving that Stk1 is a Ser/Thr protein kinase. To analyze the function of the *stk1* gene, a mutant strain lacking *stk1* was constructed. A fusion fragment (802bp) that contained upstream (453bp) and downstream (349bp) homology arms of *stk1* was amplified by PCR (Figure 1A) and cloned into pRE112 plasmid to construct the Δ*stk1* strain. In the wild-type PAO1 strain, the *stk1* gene fragment and the long gene fragment containing *stk1* were 990bp (Figure 1B, lane 1) and 1900bp (Figure 1B, lane 3), respectively. In the Δ*stk1* strain, the DNA brand at 990bp disappeared (Figure 1B, lane 2), and the band of the long gene fragment was present at 900bp (Figure 1B, lane 4). These results clearly showed that the *stk1* fragment was deleted and that the *stk1*-deletion strain (Δ*stk1*) was successfully constructed.

### Protein Quantification Analysis by LC–MS/MS

**Figure 2:**
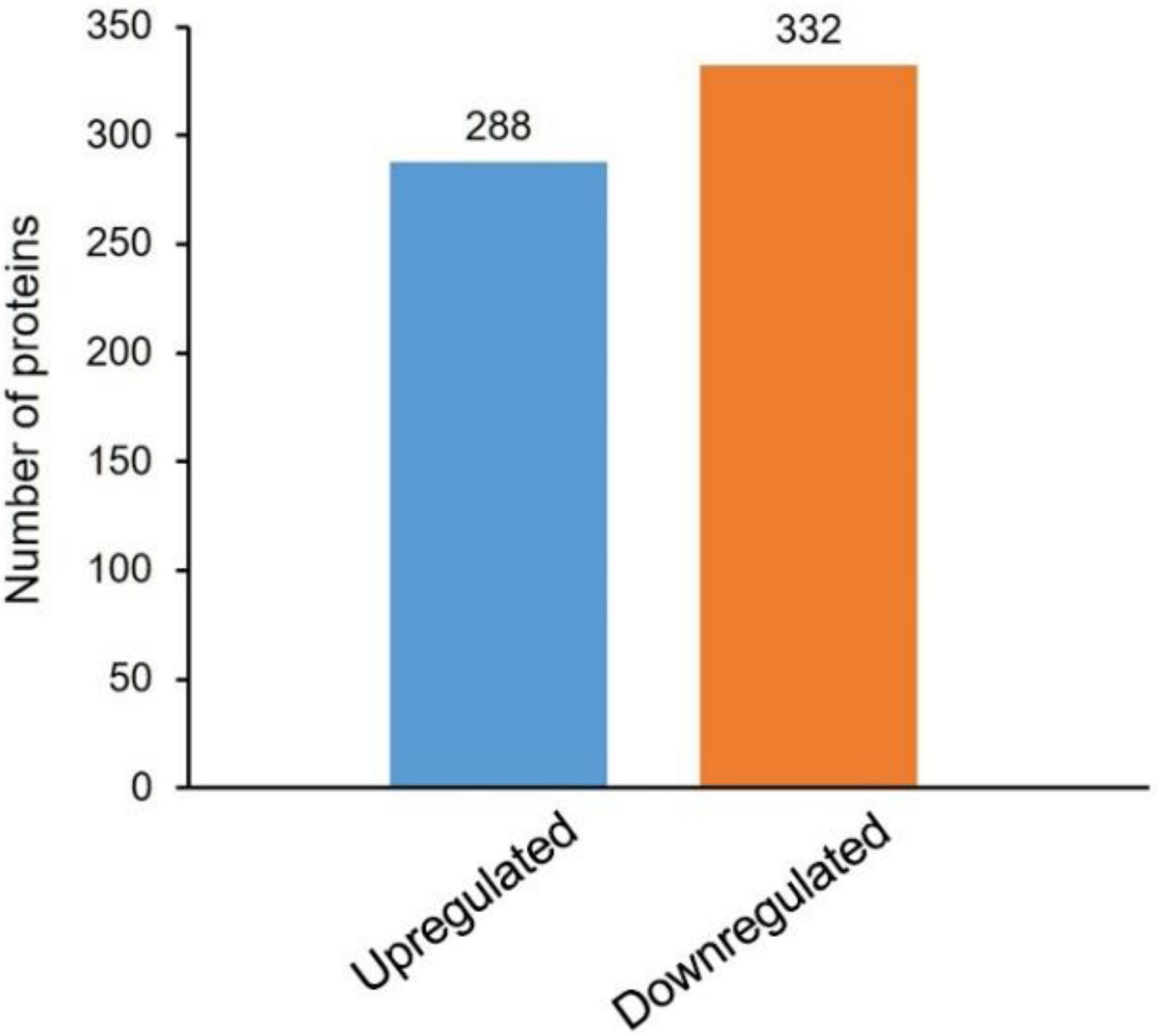
Differentially expressed proteins of ∆*stk1* compared to the PAO1 strain. The red bar represents the number of upregulated proteins and the blue bar represents the number of downregulated proteins.

TMT-based quantitative proteomics was performed to screen the differentially expressed proteins between the PAO1 and Δ*stk1* strains. After a complete technical process, a total of 316,970 secondary spectra were obtained by MS. A search of the database of MS data resulted in 57,607 available spectra, and the spectrum utilization rate was 18.2%. Based on the MS/MS spectrum database search analysis, 23,798 unique peptides were detected, corresponding to 3,525 proteins, among which 3,115 identified proteins had quantitative information in every single subject (Supplementary Table S1). The screening of differentially expressed proteins was based on whether the relative quantitative value greater than 1.3 or less than 1/1.3 and if the statistical value of *p* was less than 0.05. When the relative quantitative value was greater than 1.3, it was regarded as an upregulated protein, and when the relative quantitative value was less than 1/1.3, it was regarded as a downregulated protein. Using this standard for protein quantification, a total of 620 differentially expressed proteins were finally identified in the Δ*stk1* strain compared with the wild-type strain PAO1, among which 288 proteins were upregulated and 332 were downregulated (Figure 2; Supplementary Figure S1). This experiment was confirmed to be repeatable by the relative standard deviation (RSD; Supplementary Figure S2) and Pearson’s correlation coefficient statistical method (Supplementary Figure S3).

### GO Annotation of Differentially Expressed Proteins

In total, 620 differentially expressed proteins were characteristically and functionally annotated in detail concerning the GO, protein domain profile, KEGG pathway, and subcellular localization (Supplementary Table S2). The subcellular localization results showed that most of the upregulated proteins (67%) and downregulated proteins (60%) were distributed in the cytoplasm. Additionally, the periplasmic space accounted for 18% of the upregulated proteins and 15% of the downregulated proteins, and the proteins located in the inner membrane were 8 and 14% of the upregulated and downregulated proteins, respectively (Supplementary Figure S4; Supplementary Table S4).

**Figure 3:**
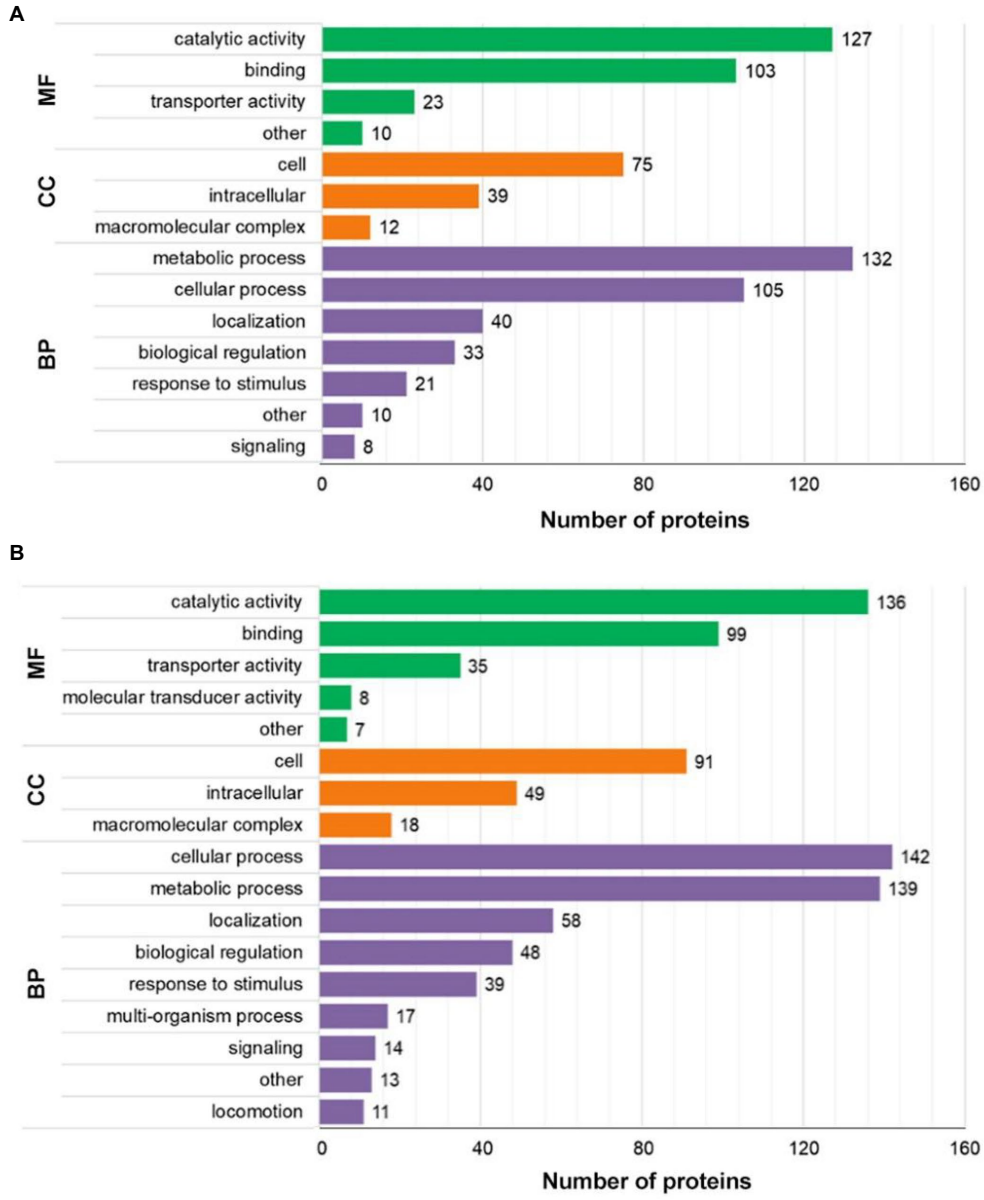
GO analysis of differentially expressed proteins. **(A)** The number of upregulated proteins categorized as molecular function, cellular component, and biological process. **(B)** The number of downregulated proteins categorized as molecular function, cellular component, and biological process. BP, biological process; CC, cellular compartment; and MF, molecular function.

Moreover, GO annotation categorized the 620 differentially expressed proteins based on molecular function, cellular component, and biological process (Figure 3; Supplementary Table S3). The upregulated proteins were classified into 14 functional groups. Among them, there were seven GO terms for biological processes, the most representative being “metabolic process” and “cellular process,” three GO terms for cellular components, the most representative being “cell” and “intracellular,” and four GO terms for molecular functions, the most representative being “binding” and “catalytic activity.” The downregulated proteins were classified into 17 functional groups, among which there were nine GO terms for biological processes, the most representative being “metabolic process” and “cellular process,” three GO terms for cellular components, the most representative being “intracellular” and “cell,” and five GO terms for molecular functions, the most representative being “catalytic activity” and “binding.”

### Enrichment Analysis of Differentially Expressed Proteins

**Figure 4:**
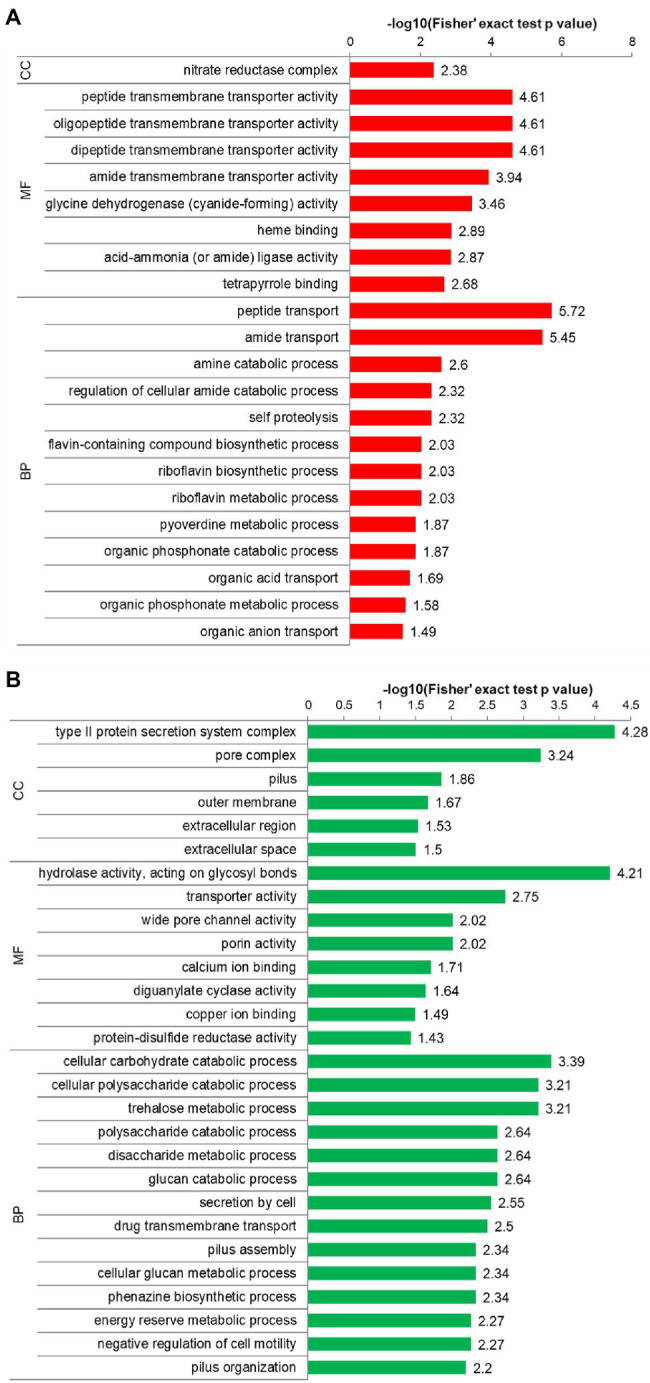
GO enrichment analysis. **(A)** GO enrichment results for upregulated proteins. **(B)** GO enrichment results for downregulated proteins. The *y* axis denotes the GO functional classification enriched by the differentially expressed protein, and the *x* axis denotes the −log_10_ of the value of *p* of Fisher’s exact test of the significance of the enrichment. BP, biological process; CC, cellular compartment; and MF, molecular function.

To detect significantly enriched biological function types, we performed a GO enrichment analysis and ranked the terms by the enrichment score. According to the GO enrichment results, the significantly enriched molecular function, cellular component, and biological process terms for the differentially expressed proteins are shown in Figure 4; Supplementary Figure S5, and Supplementary Table S5. The upregulated proteins were enriched in a wide range of molecular function terms, including the transmembrane transporter activity of peptides, oligopeptides, dipeptides, and amides. In biological process terms, the upregulated proteins were not only significantly enriched in peptide transport and amide transport (their value of *p* were twice as high as other biological processes), but also enriched in metabolic processes of some macromolecular substances, such as cellular amide, riboflavin, pyoverdine, and organic phosphonate (Figure 4A). For downregulated proteins, the enriched molecular function terms were hydrolase activity, acting on glycosyl bonds, transporter activity, wide pore channel, and porin activity, and the enriched biological process terms were catabolic processes of carbohydrates, such as cellular carbohydrate, cellular polysaccharide, disaccharide, glucan and trehalose, as well as energy reserve metabolic process (Figure 4B). In cellular components, the downregulated proteins were enriched in the type II protein secretion system complex and pore complex (Figure 4B). These results indicate that the upregulated proteins have significant roles in protein transmembrane transport and peptide transport and that the downregulated proteins are mainly involved in the catabolism of glycosides.

**Figure 5:**
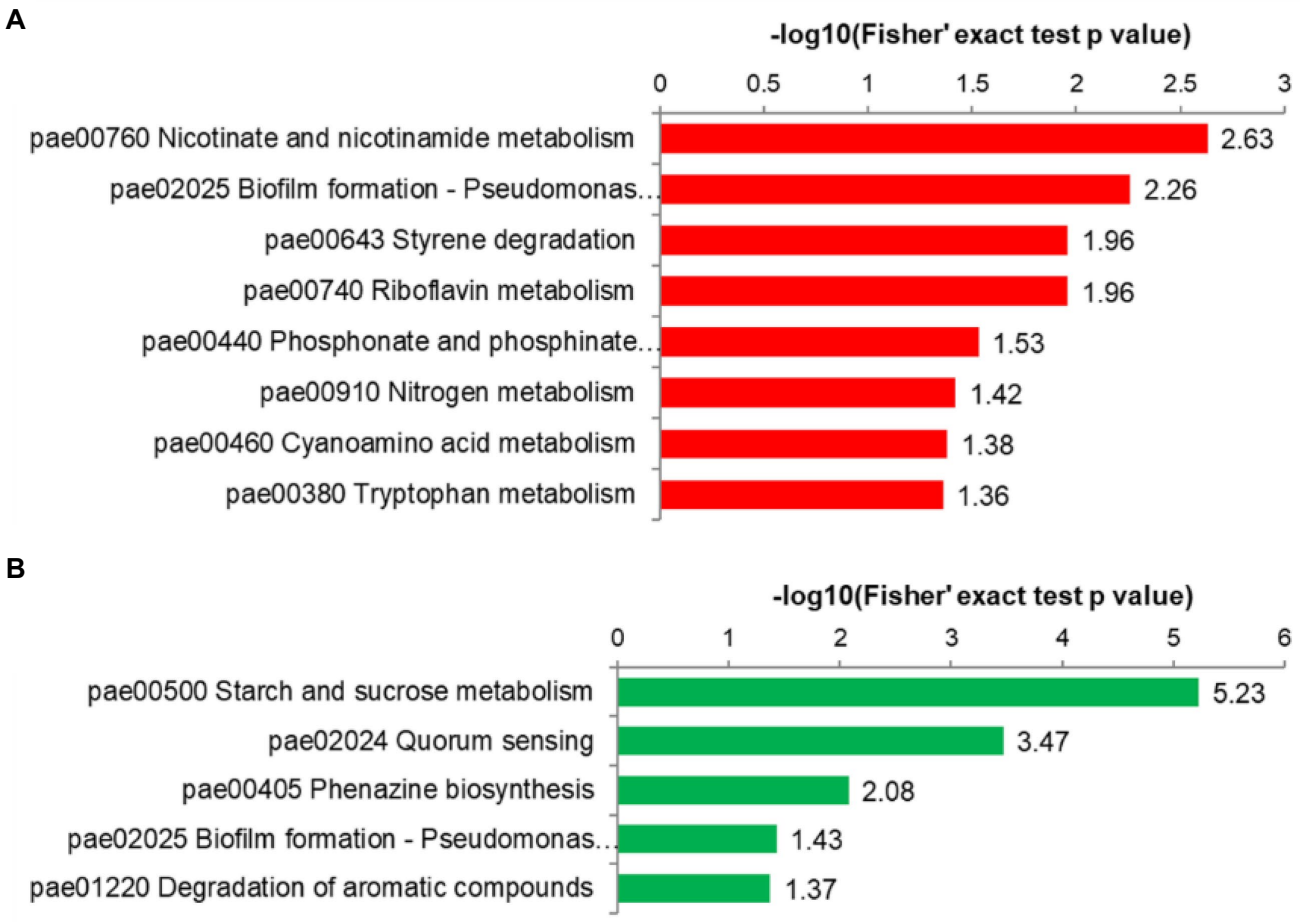
Kyoto Encyclopedia of Genes and Genomes (KEGG) pathway enrichment analysis. **(A)** KEGG pathway enrichment results for upregulated proteins. **(B)** KEGG pathway enrichment results for downregulated proteins. The *y* axis denotes the categories of KEGG pathways. The *x* axis is the −log_10_ for Fisher’s exact test of value of *p* for the significance of enrichment.

The proteins were classified by KEGG pathway enrichment analysis and fold enrichment by the bubble chart (Figure 5; Supplementary Figure S6; Supplementary Table S6). The upregulated proteins produce a total of 41 pathways and the downregulated proteins produce 42 pathways. The upregulated proteins were enriched in pae00760 nicotinate and nicotinamide metabolism, pae02025 biofilm formation, pae00643 styrene degradation, and pae00740 riboflavin metabolism (Figure 5A). Correspondingly, the downregulated proteins were enriched in pae02024 quorum sensing, pae00500 starch and sucrose metabolism, and pae00405 phenazine biosynthesis (Figure 5B). Notably, those differentially expressed proteins associated with the quorum sensing (QS) system, including the downregulated proteins of RhlR, PqsH, LecA, LasB, and PhnB, and the upregulated proteins of LasI and RhlA were found, as shown in the pae02024 quorum sensing map (Supplementary Figure S8). The QS network of *P. aeruginosa* is a multi-layered hierarchy composed of at least four interconnected signal mechanisms, including Las, Rhl, Pqs, and Iqs systems (Lee and Zhang, 2015; Turkina and Vikström, 2019; Zhou et al., 2020). These differentially expressed QS proteins are mainly involved in regulating the production of rhamnolipid and lectin A (Diggle et al., 2006; Soto-Aceves et al., 2021) and play a role in promoting the formation and maintenance of the mature structure of biofilm (Fila et al., 2018). This result indicates that Stk1 may mediate the QS system to regulate the virulence and resistance of *P. aeruginosa*.

**Figure 6:**
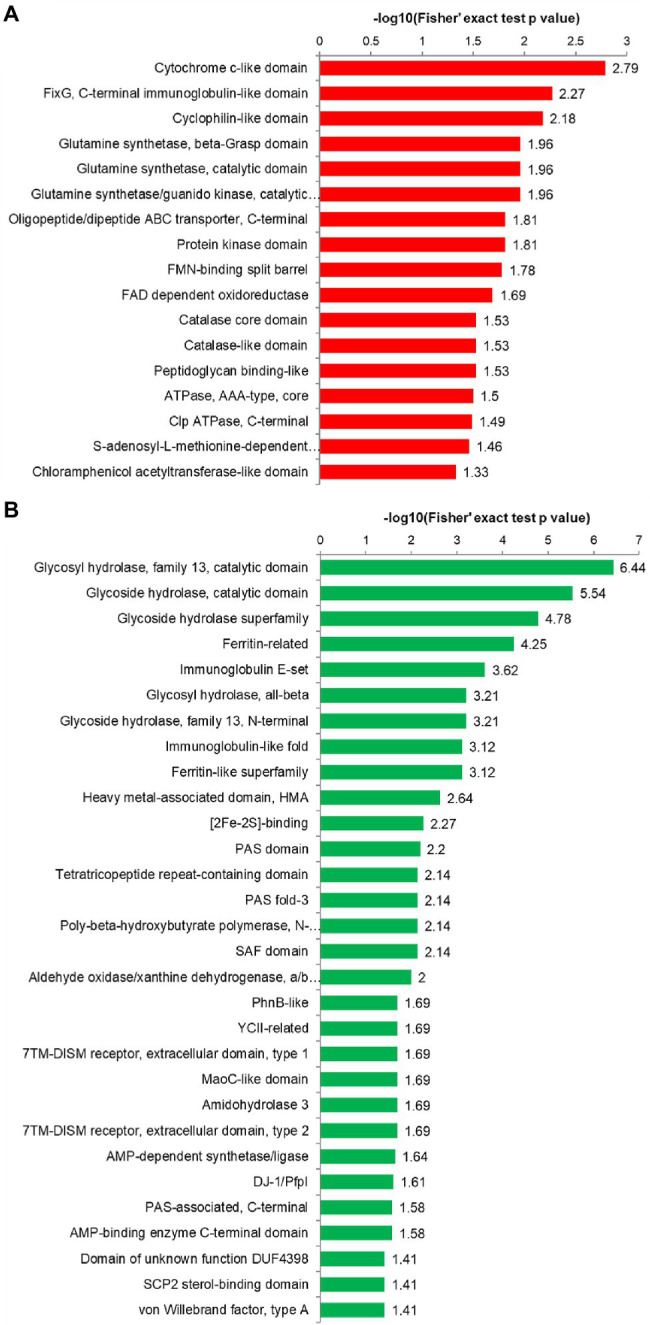
Protein domain enrichment analysis. **(A)** Protein domain enrichment results of upregulated proteins. **(B)** Protein domain enrichment results of downregulated proteins. The *y* axis denotes the categories of the protein domain. The *x* axis denotes the enrichment score [−log(value of *p*)].

Further protein domain enrichment analysis showed that upregulated proteins were significantly enriched in the cytochrome c-like domain and FixG, the C-terminal immunoglobulin-like domain, and the most enriched protein domains of the downregulated proteins were the catalytic domains of glycosyl hydrolase and glycoside hydrolase and the ferritin-related protein domain (Figure 6; Supplementary Figure S7; Supplementary Table S7). In the protein domain enrichment analysis, we found that the upregulated proteins were enriched in the protein kinase domain. Another result from the KEGG pathway analysis was that the upregulated proteins were enriched in pae00440 phosphonate and phosphinate metabolism (Figure 6A; Supplementary Figure S9). Thus, the significantly upregulated proteins were related to kinase activity and the biological process of phosphorylation, corresponding to our predicted function of Stk1.

### Functional Rich Clustering Analysis of Protein Classification Based on the Expression Quantitative Level

**Figure 7:**
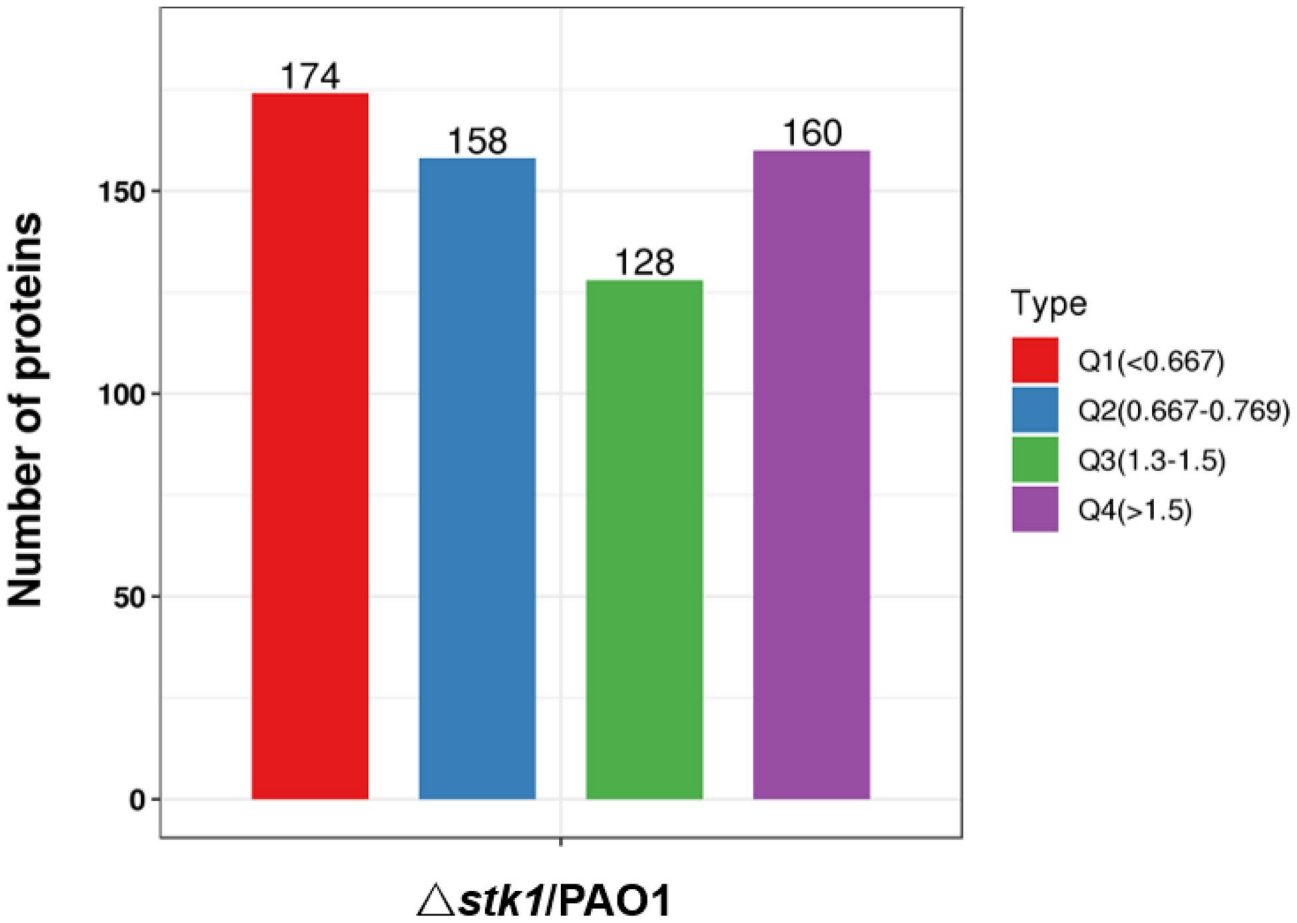
Differentially expressed protein groups based on their differential expression ratios.

**Figure 8:**
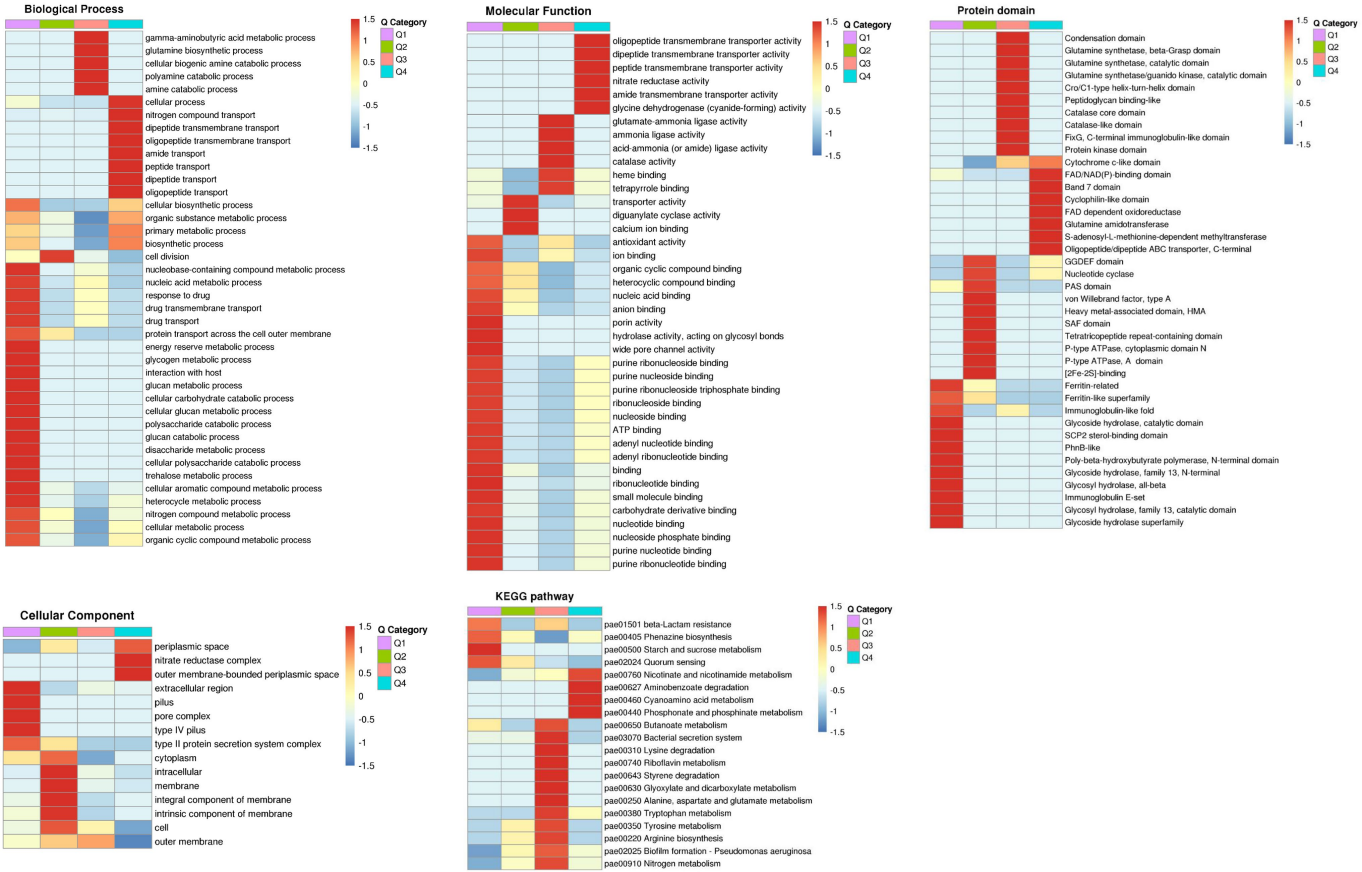
Heat maps showing the enrichment for GO (biological process, molecular function, and cellular component), the KEGG pathway, and protein domain of differentially expressed protein groups, divided based on their differential expression ratios. Red color indicates significant enrichment and blue color indicates insignificant enrichment.

For differentially expressed proteins, we performed KEGG pathway, GO, and protein domain enrichment for each comparison group and performed cluster analyses. According to their differential expression multiples, we divided them into four parts, Q1–Q4 (Figure 7), to find the correlation of protein functions with different differential expression multiples. Then, according to the value of *p* of Fisher’s exact test obtained by the enrichment analysis, the relevant functions in the different groups were grouped using the hierarchical clustering method and drawn as a heat map (Figure 8).

For KEGG pathways, 20 pathways were clustered for the four groups. Q1 and Q4 had one new term compared with the KEGG pathways enrichment analysis in each group, pae01501 β-lactam resistance and pae00627 aminobenzoate degradation, respectively. More than half of the Q3 pathways had already been identified by KEGG pathway enrichment analysis. Meanwhile, 40 protein domains were clustered, more than three-fourths of which had already been identified by protein domain enrichment analyses. The new terms found were the GGDEF domain, nucleotide cyclase, band 7 domain, condensation domain, and Cro/C1-type helix-turn-helix domain. Q1 and Q2 were mainly the protein domain enrichment results of downregulated proteins, and most of Q3 and Q4 were the protein domain enrichment results of upregulated proteins.

For GO, 15 enriched cellular component terms were found among the four groups, which included nine new terms from the GO enrichment analysis. Forty enriched biological process terms were found among four groups, and more than three-fourths of those in Q1 coincided with the GO enrichment results of downregulated proteins. There were 40 enriched molecular function terms among the four groups, almost all of which were confirmed in the GO enrichment analysis result. The functional enrichment-based clustering results for protein groups with different quantitative expression ratios were roughly similar to the enrichment analysis results. Based on the above results, we found that most biological process Q1 terms were related to biological metabolic/catabolic processes, all of which were expressed by downregulated proteins. Q3 and Q4 were both related to some transmembrane transporter activities and transport processes in the biological processes and molecular functions, which were expressed by upregulated proteins. Thus, we predicted that the function of the Stk1 kinase protein was related to catabolic processes and transmembrane transport activity.

### *stk1* Deletion Weakens the Twitching Motility

Through GO enrichment analysis and protein domain enrichment analysis, we found that many downregulated proteins are associated with type IV pilus-dependent motility and type IV pilus biogenesis. Previous reports suggested that protein appendages (mainly flagella and type IV pili) located on the cell surface play an important role in the formation of biofilms by affecting bacterial migration and solid surface attachment (Gibiansky et al., 2010; Klausen et al., 2010). *Pseudomonas aeruginosa* can successfully establish chronic infection in patients with cystic fibrosis. The first step in the infection process is epithelial adhesion and colonization, which is mediated to a certain extent by a type IV pilus (Drr et al., 2010). Besides host tissue adhesion, type IV pilus promotes biological functions important for bacterial pathogenicity, such as twitching motility and DNA uptake (Craig et al., 2004). *In vitro* and *in vivo* studies showed that mutants lacking functional type IV pili have a significant reduction in colonization, biofilm formation, and ability to spread (O’toole and Kolter, 1998; Wozniak and Keyser, 2004; Li et al., 2016; Nieto et al., 2019).

**Figure 9:**
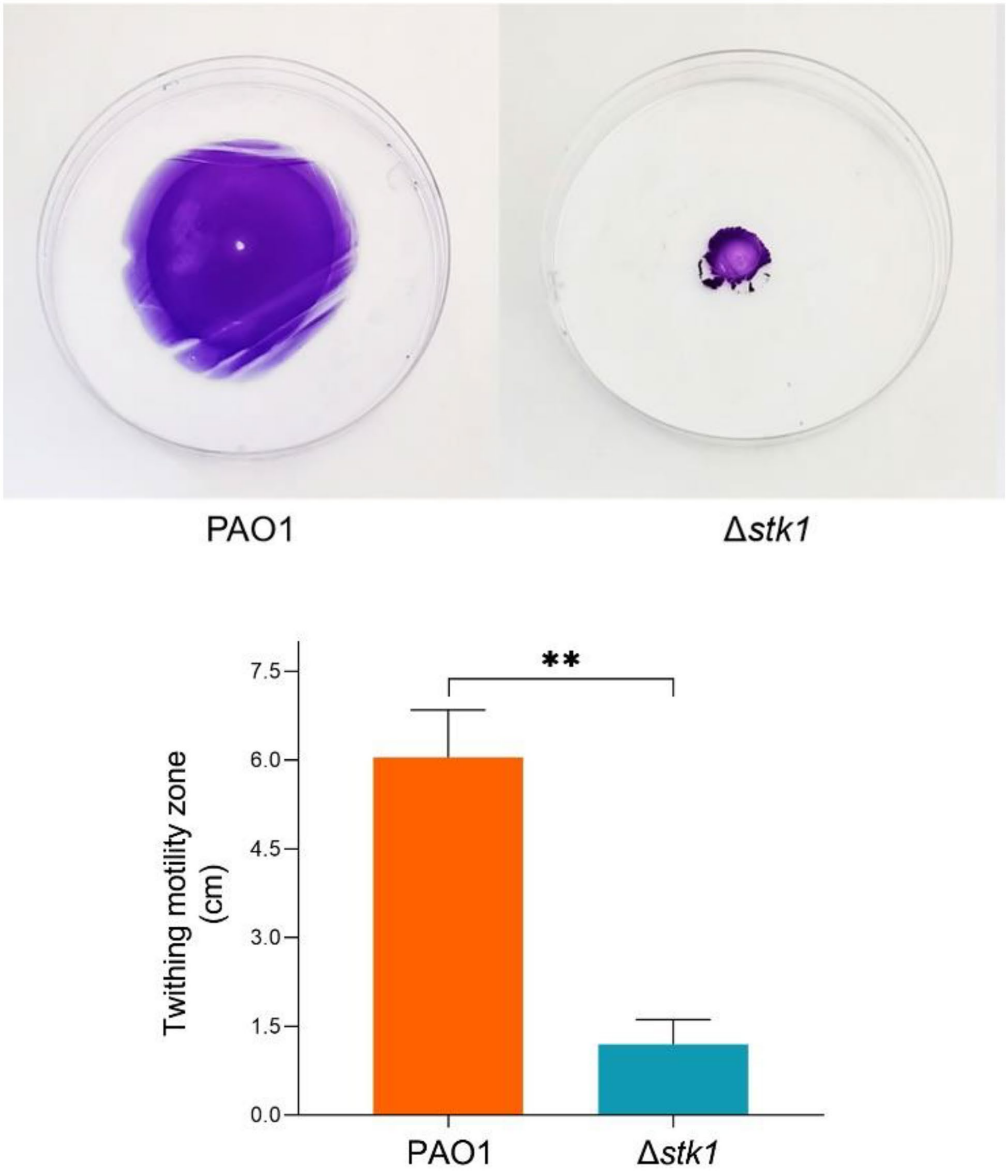
The *stk1* deletion attenuates the function of the type IV pilus. Detecting the effect of twitching motility can reflect whether the function of type IV fimbriae functions normally. The PAO1 and Δ*stk1* strains were both cultured overnight and then added dropwise to the middle of the medium. They were incubated at 37°C for 24h and then stained with 1% crystal violet solution for 15min. Twitching motility diameters were measured. The data represent the average values as the means (SD) from three independent experiments (^**^ *p*<0.01).

Twitching motility is a flagellum-independent mode of surface translocation mediated by a type IV pilus. Its basic function is to help bacteria explore the surface to which it attaches, and it is also a means for bacteria to escape from the surface under certain conditions. For example, early studies showed that the cyclic disulfide at the carboxyl end of PilA plays a role in adhesion. It can use the main chain atoms to bind to the receptor on the surface of epithelial cells (Wong et al., 1995; Audette et al., 2004), which is essential for the adhesion and detachment of bacteria from the surface (Conrad et al., 2011). Bacterial twitching motility is mainly powered by three physiological processes: pilus assembly, adhesion of the top protein of the pilus to the surface, and pilus contraction. The loss or weakening of twitching motility in these three processes may create one or more problems. Therefore, it may be a therapeutic strategy to destroy the function of type IV pili and cut off the interaction with host cells in specific ways. In our research, several type IV pilus function-related proteins were downregulated in the Δ*stk1* strain compared with the wild-type PAO1 strain (Table 2). In *P. aeruginosa*, the FimU promoter is located upstream of six open reading frames (*fimU-pilVWXY1E*) and is responsible for encoding the precursor protein required for normal tissue assembly and function of the type IV pilus and the calcium ion-dependent contractile protein (Belete et al., 2008). Moreover, a study found that phosphorylation of the response regulator AlgR resulted in direct activation of the *fimU-pilVWXY1Y2E* operon, which was required for the assembly and export of a functional type IV pilus (Whitchurch et al., 2002). Additionally, the sigma factor AlgT and a high level of transcriptional regulator AmrZ inhibit twitching motility (Xu et al., 2021). This shows that the twitching motility mediated by type IV pili is regulated in many ways. The crystal violet staining results showed that there was a significant difference in the colony area of Δ*stk1* on the bottom surface of the culture dish compared with that of the wild-type strain PAO1 (*p*<0.01; Figure 9). Δ*stk1* lost twitching motility, indicating that the function of the type IV pilus was affected. This result demonstrates, as a Ser/Thr protein kinase, Stk1 may modulate twitching motility through the phosphorylation of proteins that mediate pilus adhesion or contractile function.

**Table 2:** The downregulated type IV pilus-related proteins in the Δ*stk1* strain compared with the PAO1 strain.

Protein accession	Protein description	GO term description	Gene name	Δ*stk1*/PAO1 ratio
G3XCZ0	Type 4 fimbrial biogenesis protein	Type IV pilus-dependent Motility pilus assembly	*fimU*	0.553
G3XD15	Type 4 fimbrial biogenesis protein	Type IV pilus-dependent motility pilus assembly	*pilW*	0.501
G3XD84	Type 4 fimbrial biogenesis protein	Type IV pilus-dependent motility pilus assembly	*pilV*	0.306
Q9HVM8	Type IV pilus biogenesis factor	Type IV pilus-dependent motility	*pilY1*	0.688
Q9HXJ2	Type IV pilus assembly protein	Type IV pilus-dependent motility pilus assembly	*pilF*	0.693
P22610	Prepilin leader peptidase/*N*-methyltransferase	Type IV pilus biogenesis pilus assembly	*pilD*	0.478
G3XCY4	Transcription factor	Type IV pilus-dependent motility	*amrZ*	0.452

### *stk1* Deletion Promotes a Growth Competition Advantage

In the GO and KEGG enrichment analyses, 288 upregulated proteins were identified, among which four type VI secretion system H1-related proteins were enriched in the pae03070 bacterial secretion system (Table 3; Supplementary Figure S10).

**Table 3:** The differentially expressed T6SS-H1-related proteins in the Δ*stk1* strain compared with the PAO1 strain.

Protein accession	Protein description	Gene name	Δ*stk1*/PAO1 ratio
Q9I749	Type VI secretion system sheath protein	*tssB1*	1.611
Q9I748	Type VI secretion system sheath protein	*tssC1*	1.379
Q9I746	Type VI secretion system accessory component	*tagJ*	1.375
Q9I737	Type VI secretion system spike protein	*vgrG1b*	1.363

In gram-negative pathogens, six different types of protein secretion systems have been found to play important roles in communication with the cellular environment. The type VI secretion system (T6SS) is one of the most recently discovered secretion systems and is distributed widely in gram-negative bacterial species, including *P. aeruginosa* (Chen et al., 2015; Pena et al., 2019). *Pseudomonas aeruginosa* encodes three sets of independent T6SSs, namely, H1-, H2-, and H3-T6SS. These can directly inject effector proteins into host target cells to perform their specific biological functions, which is beneficial to the survival of *P. aeruginosa* (Cianfanelli et al., 2016; Sana et al., 2016; Chen et al., 2020). The tail of the T6SS is a puncture device similar to bacteriophages, which is composed of TssB/C and contains a stack of Hcp tubes (Lossi et al., 2013; Forster et al., 2014). The top of the Hcp tube is a puncture device composed of VgrG. *Pseudomonas aeruginosa* employs the T6SS to deliver toxic antimicrobial and antieukaryotic effectors to target cells. The T6SS not only mediates biofilm formation, metal ion uptake, and interaction with eukaryotic host cells but also plays an important role in the competition between bacteria (Records, 2011; Wang et al., 2020) Previous studies have shown that the H1-T6SS of *P. aeruginosa* can protect cells from exogenous DNA transfer mediated by rp4 coupling (Roux et al., 2015), some effectors of the gene encoding the toxin TSE7 are dependent on VgrG1b and H1-T6SS (Pissaridou et al., 2018), and H3-T6SS contributes to the interbacterial competitive fitness of *P. aeruginosa via* delivery of the toxin PldB to host cells (Jiang et al., 2014). The T6SS can often give a competitive advantage in growth during *P. aeruginosa* survival in the same environment as other bacteria (Lin et al., 2015). More importantly, *P. aeruginosa* uses T6SS to secrete toxic proteins that can inhibit the growth of other bacteria or even kill them to gain a competitive advantage (Hood et al., 2010; Jiang et al., 2014; Wood et al., 2019).

**Figure 10:**
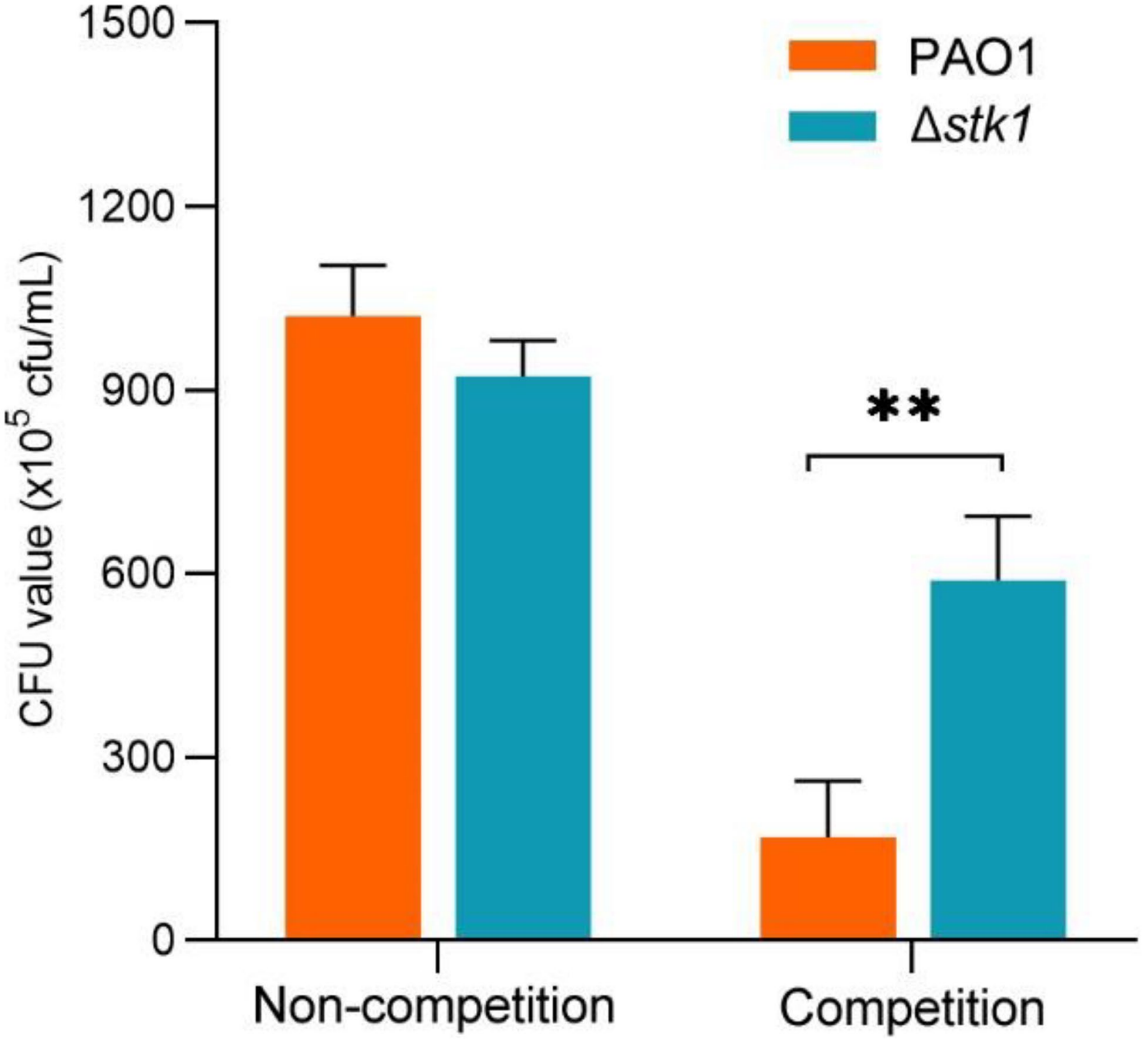
*stk1* deletion provides a growth competition advantage against *Escherichia coli* K12. Δ*stk1* or wild-type PAO1 was mixed 1:1 with *E. coli* K12 as the competition group. Additionally, the growth of PAO1 and Δ*stk1* in liquid LB medium alone was used as the noncompetition control. After incubating at 37°C with shaking for 6h, the cultures were spread on selective cetrimide agar plates at an appropriate dilution ratio. The CFU values were determined by counting the bacterial colonies. The bars represent the mean CFU value (SD) from three independent experiments (^**^ *p*<0.01).

Hence, we conducted growth competition experiments *in vitro* using the PAO1 and *E. coli* K12 or Δ*stk1* and *E. coli* K12 strains. The results showed that the Δ*stk1* strain presented a significant growth competition advantage compared with the PAO1 strain (Figure 10). We speculated that the *stk1* deletion upregulated T6SS-H1-related proteins, providing a growth competition advantage.

### Confirmation of Changes in Differentially Expressed Proteins

**Figure 11:**
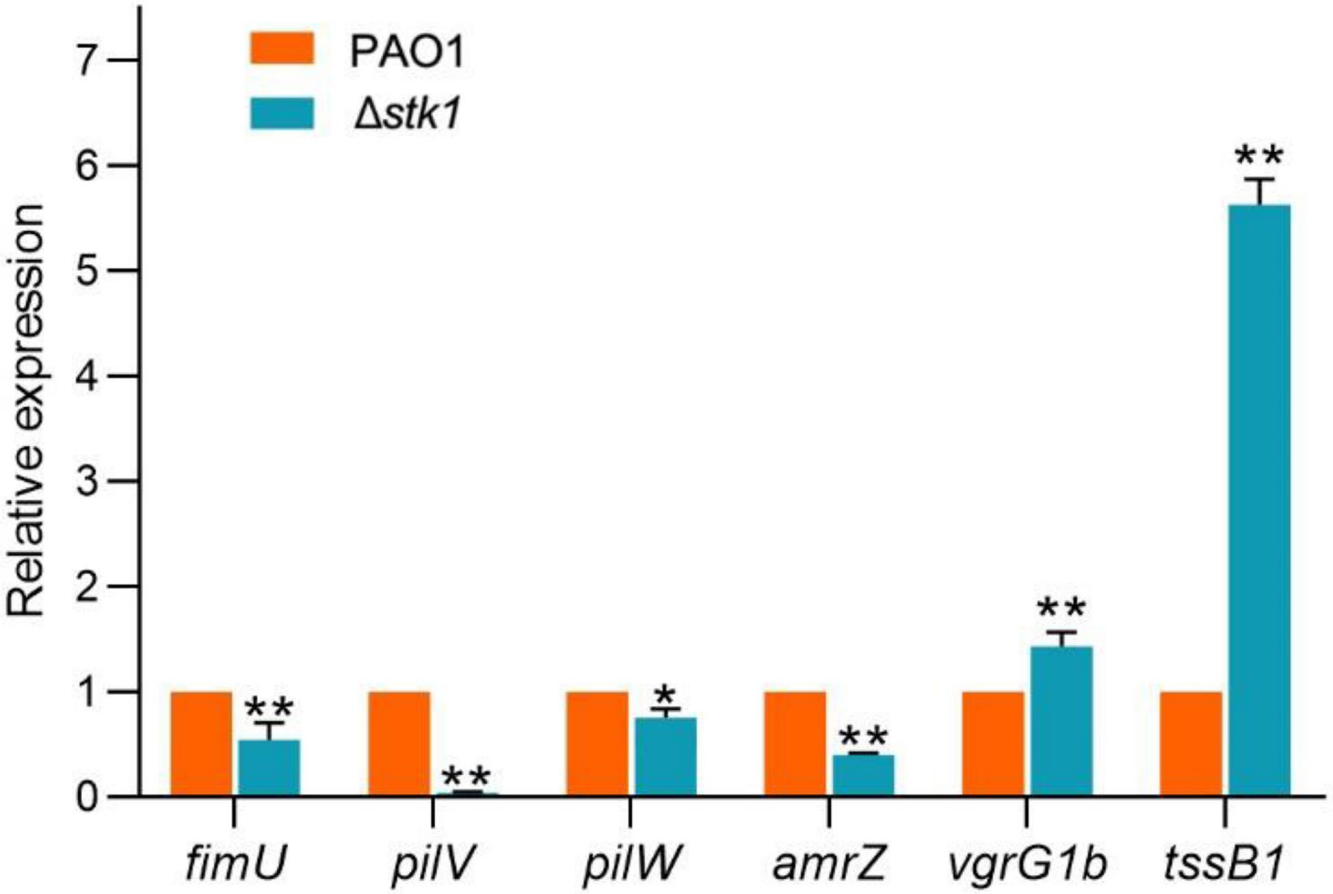
Relative expression of several differentially expressed proteins (Δ*stk1*/PAO1) at the mRNA level determined by qPCR. The data are expressed as the means (SD). The asterisks on the bars indicate that the results were significantly different from the results obtained with the wild-type strain (^*^ *p*<0.05 and ^**^ *p*<0.01).

The results from the twitching motility assay and growth competition assays both agreed with the expressed regulation trends of the type IV pilus function-related differentially expressed proteins and T6SS-H1-related differentially expressed proteins obtained by quantitative proteomics. Based on the results, we selected several proteins among the above differentially expressed proteins to further identify their expression at the mRNA level by qPCR. The results showed that four type IV pilus function-related proteins were downregulated and two T6SS-H1-related proteins were upregulated in the Δ*stk1* strain compared with the wild-type PAO1 strain (Figure 11). The change trends were consistent with the proteomics results, although the specific ratios were not always consistent. The disproportionate changes may be due to technical differences (qPCR measurement is not strictly quantitative) combined with the fact that the level of mRNA change may not directly be translated into a change of protein level, as described previously (de Godoy et al., 2008). This work further confirmed that the above results were reliable.

## Conclusion

In this study, a highly sensitive and accurate proteomic method based on TMT labeling and LC–MS/MS was used to characterize the differentially expressed proteins between the PAO1 and Δ*stk1* strains. In total, 620 differentially expressed proteins were identified, including 288 upregulated proteins and 332 downregulated proteins. Bioinformatics analyses showed that most of these differentially expressed proteins were distributed in the cytoplasm and were involved in many biological processes, such as metabolic processes, cellular processes, and catalytic activity. The enrichment analysis showed that the downregulated proteins were mainly involved in starch and sucrose metabolism, while the upregulated proteins were mainly associated with nicotinate and nicotinamide metabolism, biofilm formation, and transmembrane transport. Moreover, several downregulated proteins related to the type IV pilus and upregulated proteins related to T6SS-H1 were found in ∆*stk1* compared with PAO1, and relevant experiments were performed on these differentially expressed proteins. The alteration of these proteins was confirmed by a qPCR analysis at the mRNA level. Further experiments indicated that the deletion of *stk*1 weakens bacterial twitching motility and promotes a growth competition advantage. The impacts on these physiological roles are consistent with the changes in expression of these differentially expressed proteins identified both by proteomics and qPCR.

It is known to us that the increase of bacterial resistance has led to difficulties in clinic treatment. An important strategy to control *P. aeruginosa* infection is to develop new effectively antibiotics, but at present, it is very difficult to develop new antibiotics that are effective and are not prone to resistance (Malléa et al., 2003; Kalia and Purohit, 2011). In recent years, there have been many studies on bacterial protein phosphorylation, and the results show that phosphorylation plays an important regulatory role in bacterial virulence and resistance. As a Ser/Thr protein kinase that mediates the phosphorylation of proteins, Stk1 is involved in the regulation of a variety of signaling pathways and biological processes in *P. aeruginosa*. Although the detailed regulatory mechanisms of Stk1 still need to be revealed, our systematic analysis of the proteome of the Δ*stk1* strain provides supporting data for the study of virulence regulation, intracellular signal transduction, energy transfer, nutrient catabolism pathways, transmembrane transport, biofilm synthesis, etc. To further determine the phosphorylated substrate protein of Stk1, analyze its role in phosphorylation signal transduction, and find the relationship between phosphorylation and cellular processes, may provide a promising target for the developing new potential strategy for controlling *P. aeruginosa*.

## Data Availability Statement

The mass spectrometry proteomics data had been deposited to the ProteomeXchange Consortium *via* the PRIDE (Perez-Riverol et al., 2019) partner repository with the dataset identifier PXD027148 (http://www.ebi.ac.uk/pride/archive/projects/PXD027148).

## Author Contributions

JP conceived and designed the study. XZ, CF, and LZ performed the proteomics analysis and acquired the data. XZ and JP analyzed the data and wrote the manuscript. XZ, ZL, and YZ performed the biochemistry experiments. XZ prepared the figures. All authors contributed to the article and approved the submitted version.

## Funding

This work was supported by grants from the National Natural Science Foundation of China (31770141) and the 521 Talent Program of Zhejiang Sci-Tech University, China, to JP.

## Conflict of Interest

The authors declare that the research was conducted in the absence of any commercial or financial relationships that could be construed as a potential conflict of interest.

## Publisher’s Note

All claims expressed in this article are solely those of the authors and do not necessarily represent those of their affiliated organizations, or those of the publisher, the editors and the reviewers. Any product that may be evaluated in this article, or claim that may be made by its manufacturer, is not guaranteed or endorsed by the publisher.

## Footnotes

^1^ http://www.pseudomonas.com

^2^ http://www.ebi.ac.uk/GOA

^3^ http://www.kegg.jp/kegg/kegg1.html

^4^ http://www.ebi.ac.uk/interpro/

^5^ https://wolfpsort.hgc.jp
