# Supplementary material for: Impacts of Ser/Thr Protein Kinase Stk1 on the Proteome, Twitching Motility, and Competitive Advantage in Pseudomonas aeruginosa
Source: Front Microbiol. 2021 Sep 22;12:738690. doi: 10.3389/fmicb.2021.738690 (PMC8560001; doi:10.3389/fmicb.2021.738690)
Supplement: Supplementary file 2 [file Data_Sheet_2.docx]

**Supplementary Material**

**Impacts of Ser/Thr Protein Kinase Stk1 on the Proteome, Twitching Motility, and Competitive Advantage in *Pseudomonas aeruginosa***

Xuan Zhu^1^, Chao Feng^1^, Lantian Zhou^1^, Zhenzhen Li^1^, Yue Zhang^1^, Jianyi Pan^1^*

^1^*Zhejiang Provincial Key Laboratory of Silkworm Bioreactor and Biomedicine, College of Life Sciences and Medicine, Zhejiang Sci-Tech University, Hangzhou, China*

**Running title：**Impacts of Stk1 on *P. aeruginosa*

***Corresponding author:**

Dr. Jianyi Pan, College of Life Sciences and Medicine, Zhejiang Sci-Tech University, Hangzhou, China. E-mail: jianyi.pan@zstu.edu.cn.

**Supplementary Figure Legends**

**Fig. S1**: The volcano chart shows the statistics of the quantitative results of protein changes. The x-axis refers to the difference multiple, the y-axis refers to the significance (-log10(P value)). The orange dots indicate upregulated proteins, the green dots indicate downregulated proteins, and the gray dots indicate proteins with no difference.

**Fig. S2**: Box plot shows relative standard deviation (RSD) distribution of protein quantitative values of repeated protein samples.

**Fig. S3**: Heat map shows Pearson's correlation coefficient for protein quantification between three replicate samples.

**Fig. S4**: Subcellular localization distribution of the differentially expressed proteins. (A) Upregulated proteins; (B) Downregulated proteins.

**Fig. S5**: The bubble charts show the fold enrichment for GO analyses of the differentially expressed proteins in terms of biological process, cellular component and molecular function.

**Fig. S6**: The bubble chart shows the fold enrichment of KEGG pathways of the differentially expressed proteins.

**Fig. S7**: The bubble chart shows the fold enrichment of the protein domains of the differentially expressed proteins.

**Fig. S8**: Pathway map of the pae02024 quorum sensing enriched in differentially expressed proteins by KEGG pathway enrichment analysis. The red box indicates upregulated proteins and the green box indicates down-regulated proteins.

**Fig. S9**: Pathway map of the pae00440 phosphonate and phosphinate metabolism. The red box indicates upregulated proteins and the green box indicates down-regulated proteins.

**Fig. S10**: Pathway map of the pae03070 bacterial secretion system enriched in differentially expressed proteins by KEGG pathway enrichment analysis. The red box indicates upregulated proteins and the green box indicates down-regulated proteins.

**Supplemental Figures**


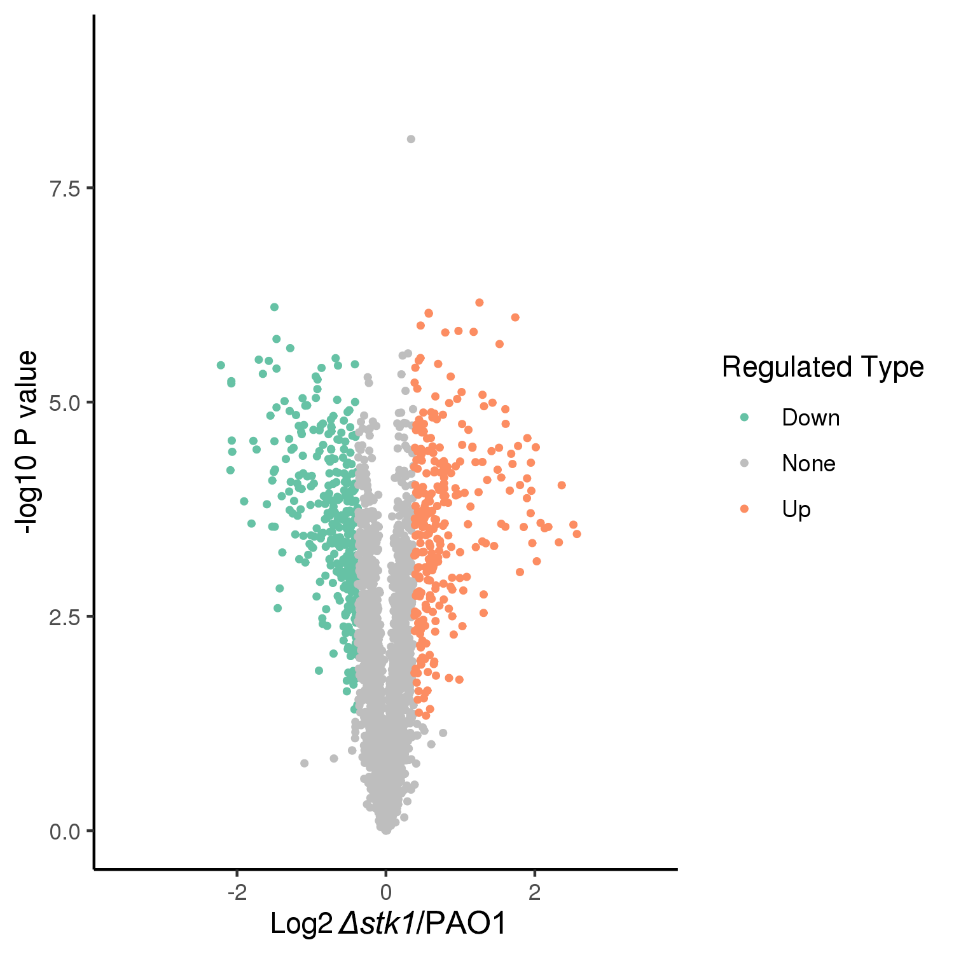


**Fig. S1**


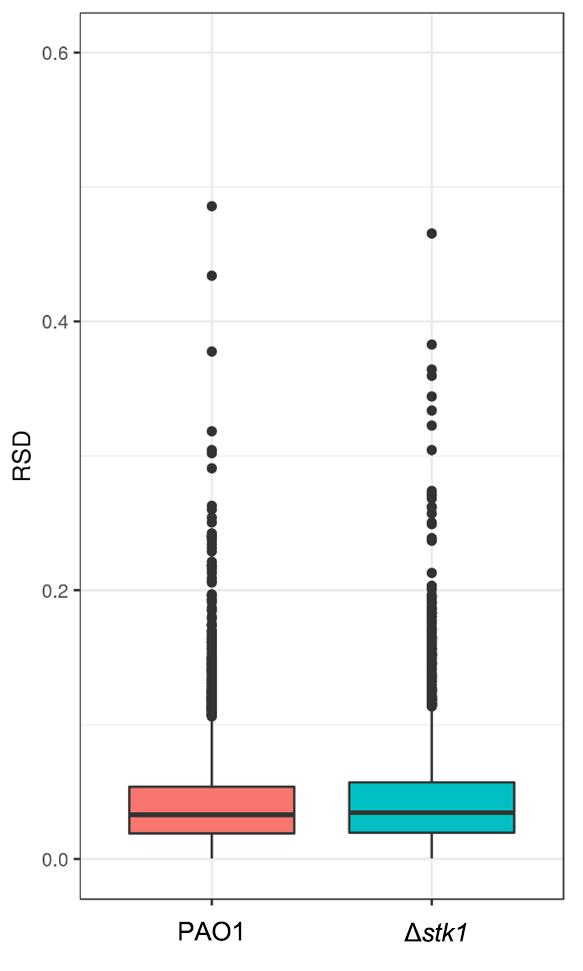


**Fig. S2**


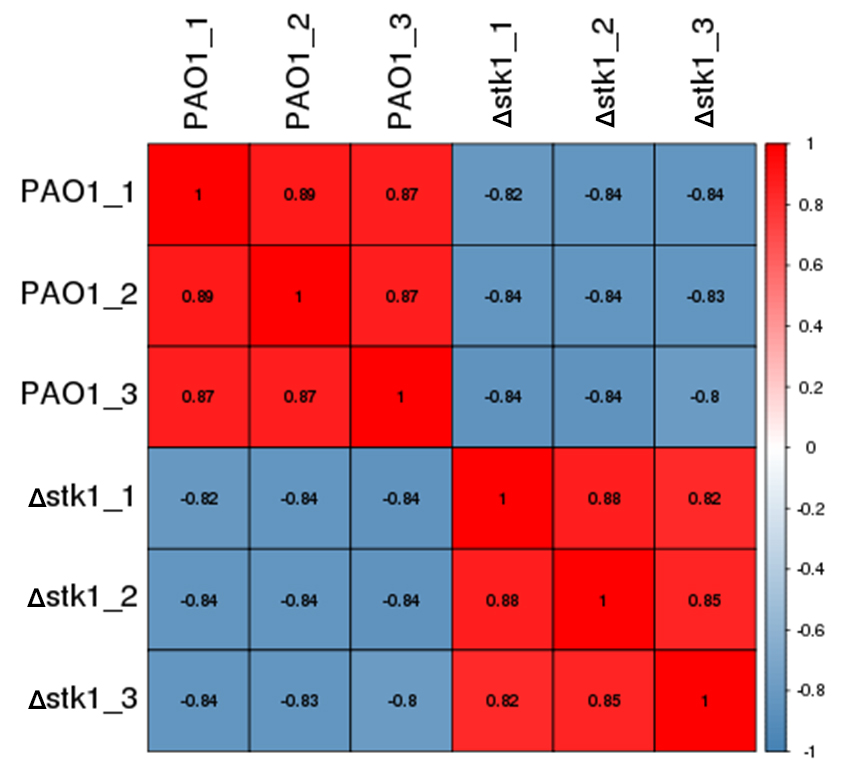


**Fig. S3**


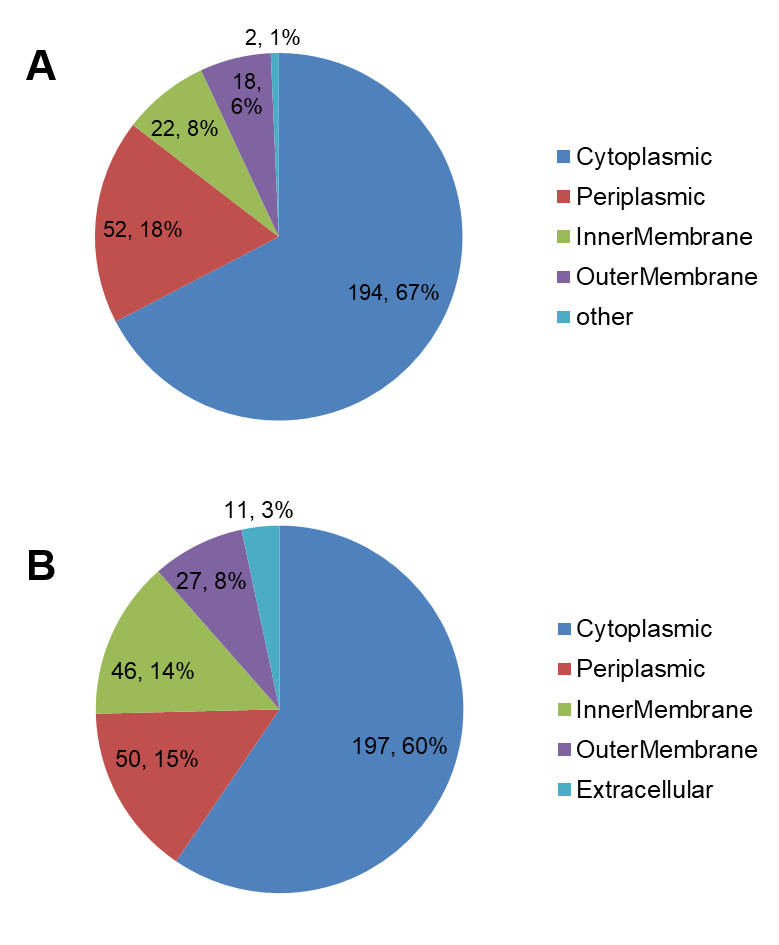


**Fig. S4**


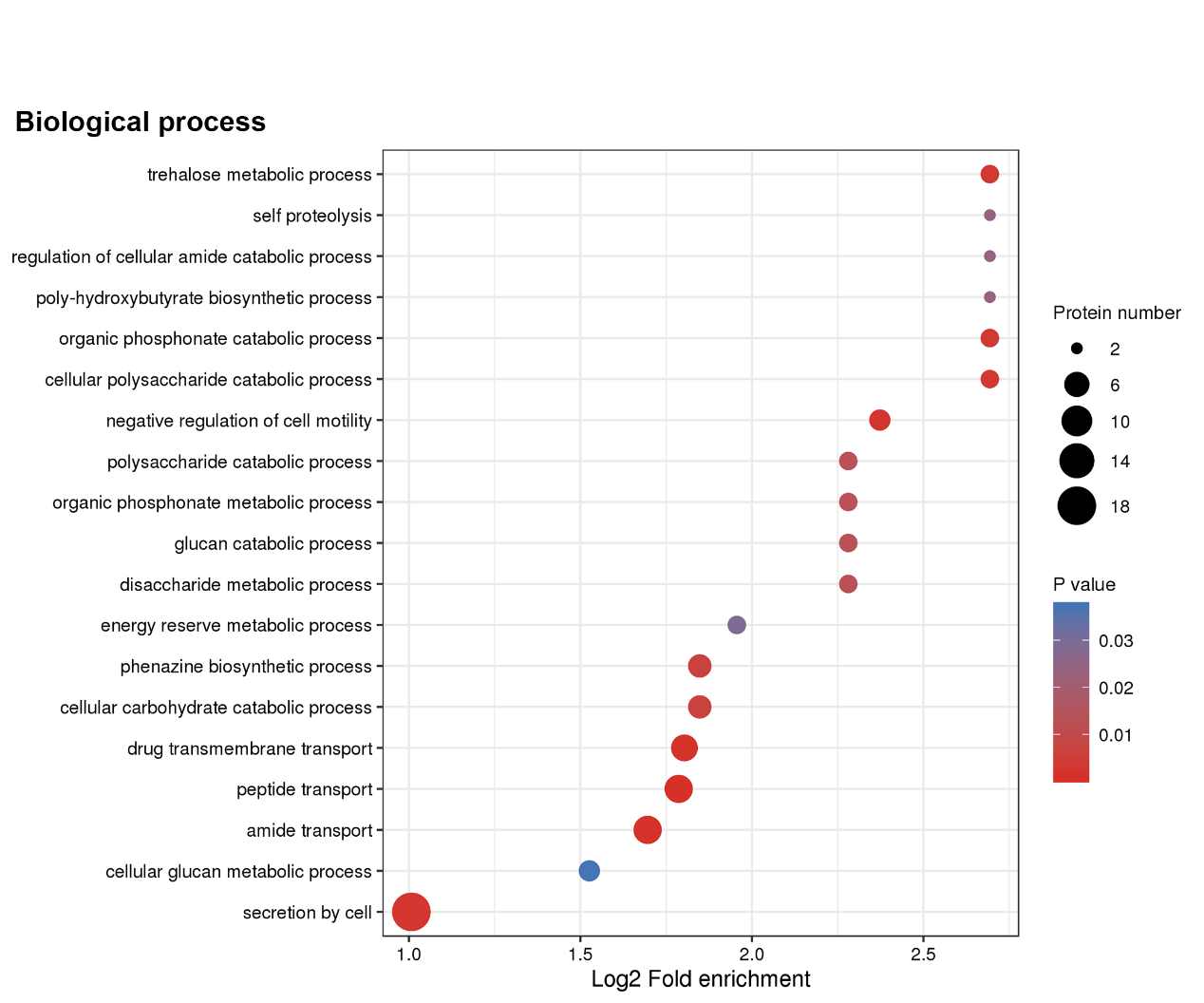

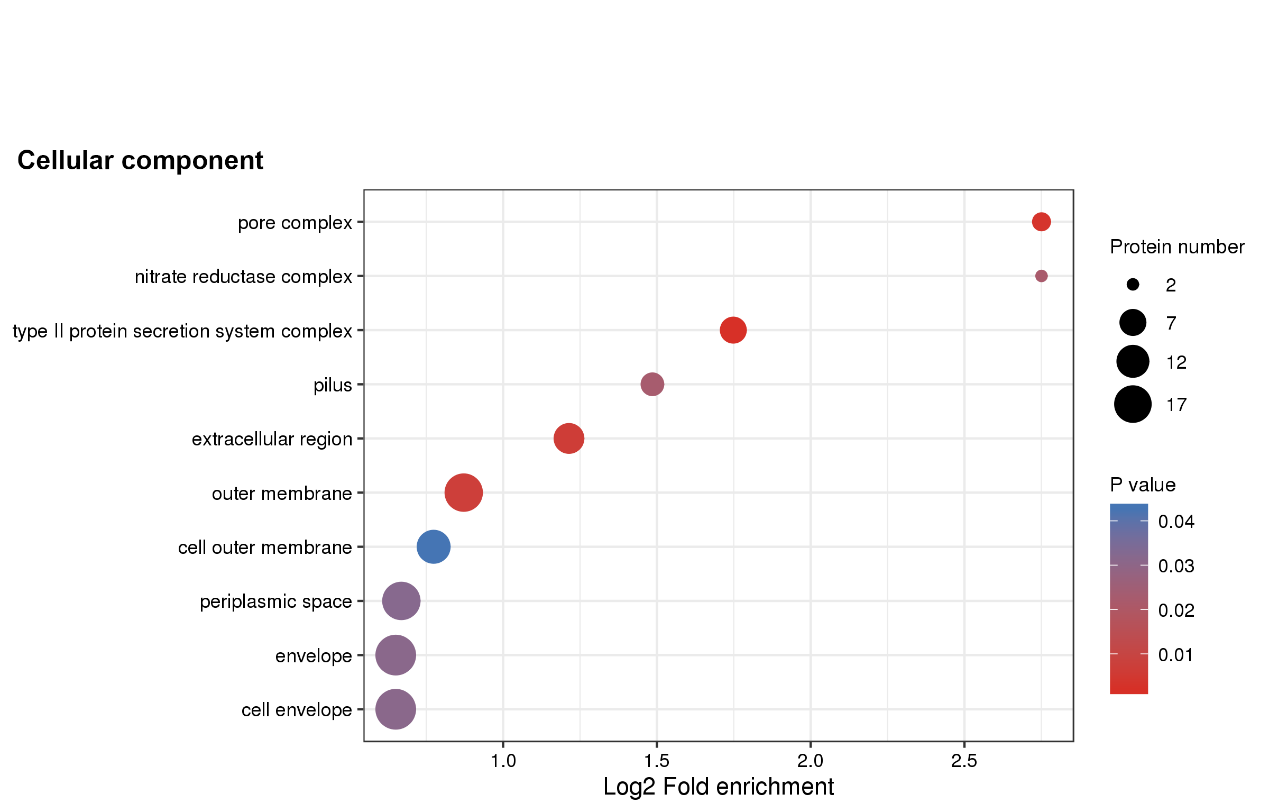

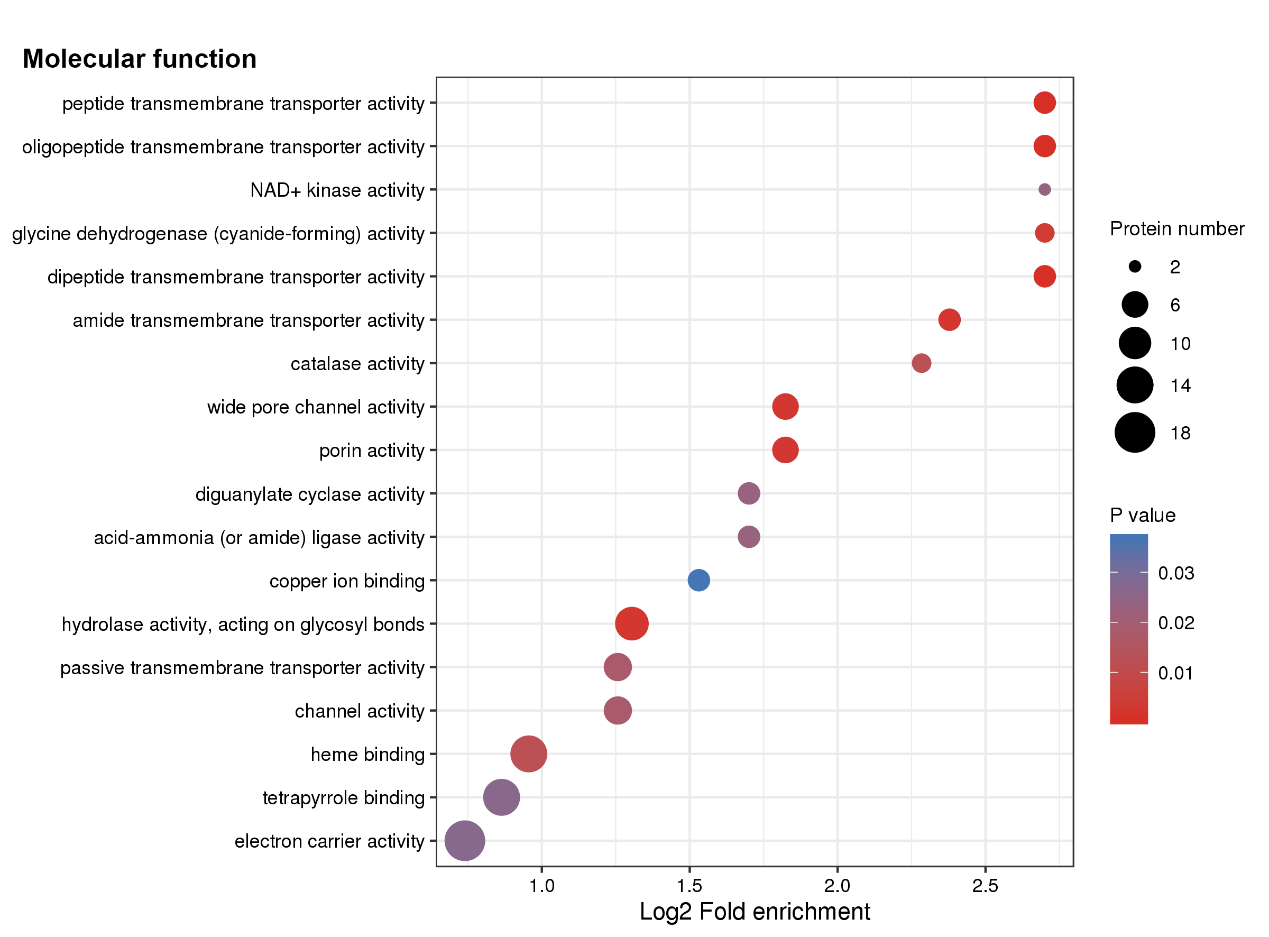


**Fig. S5**


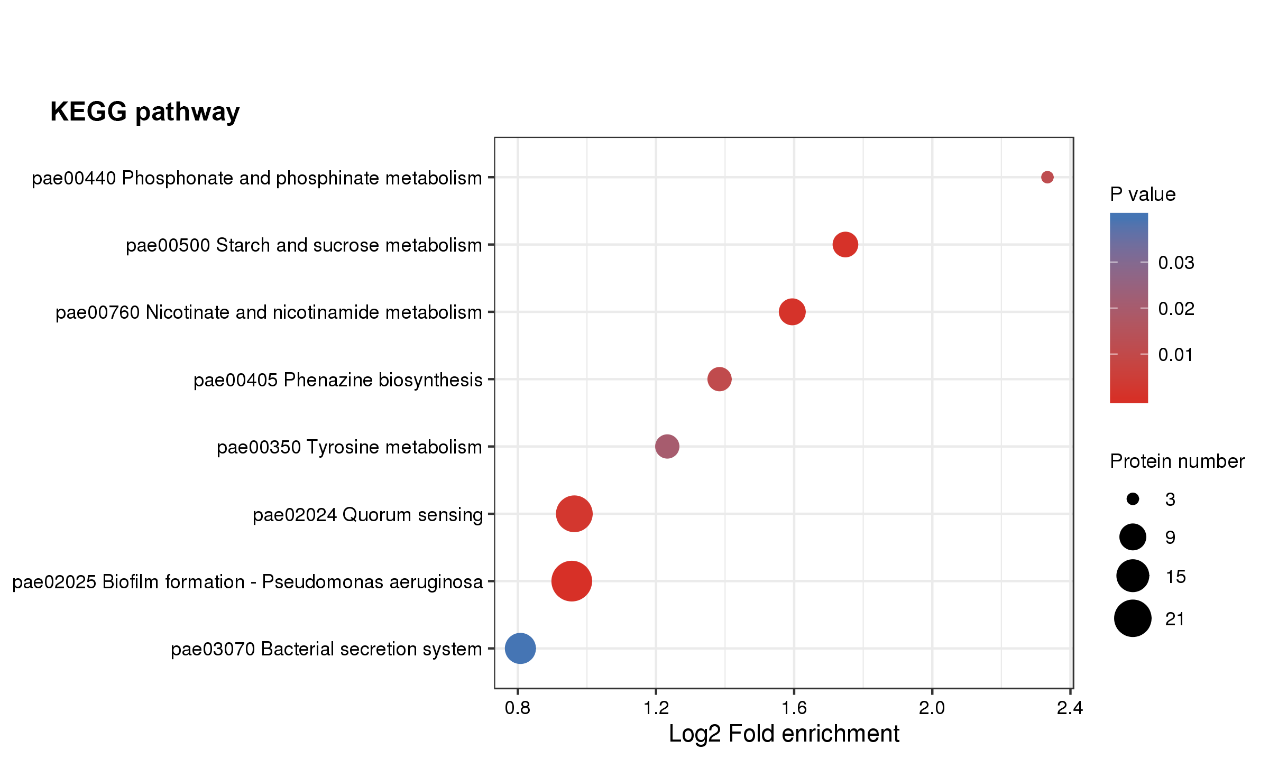


**Fig. S6**


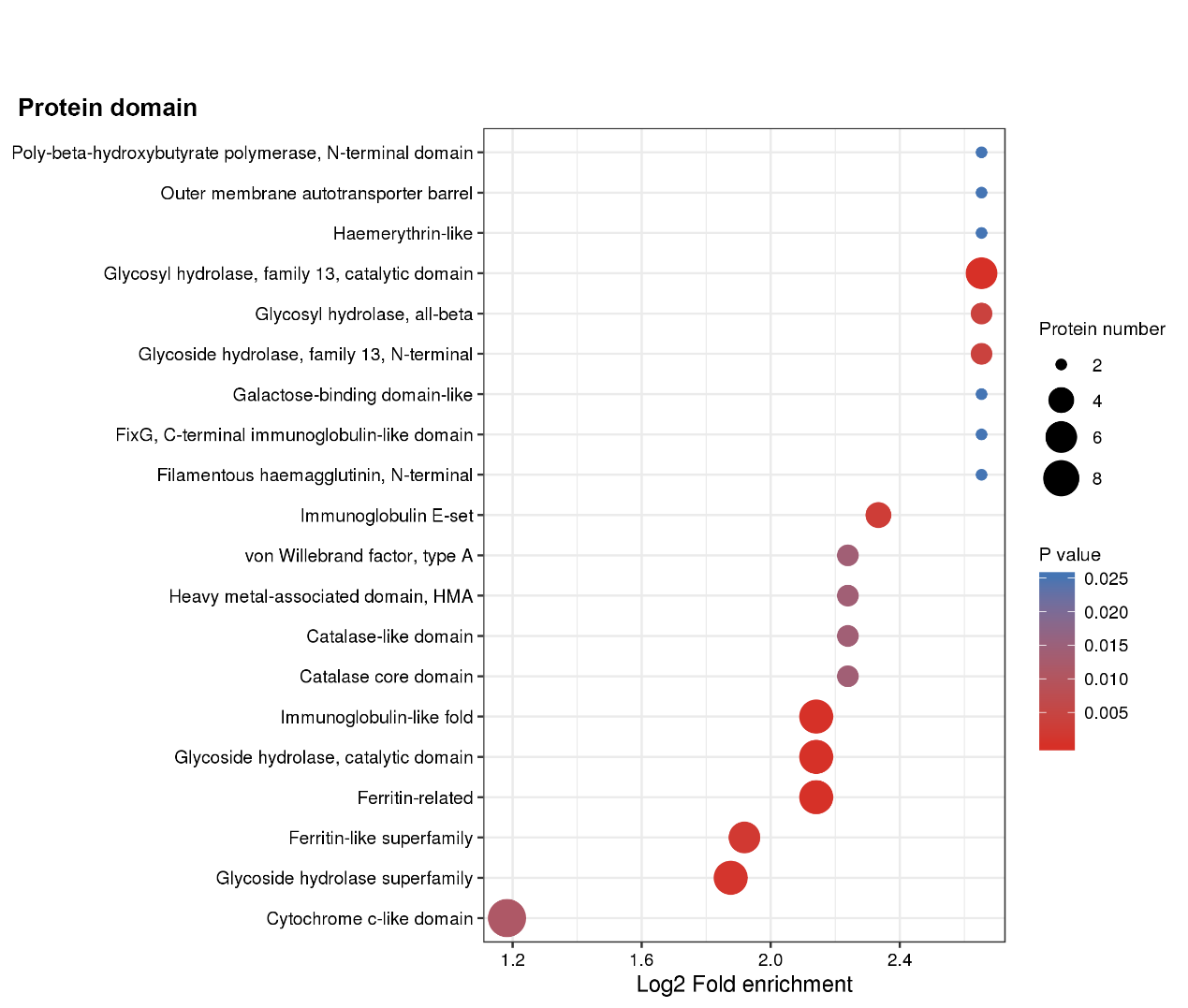


**Fig. S7**

**
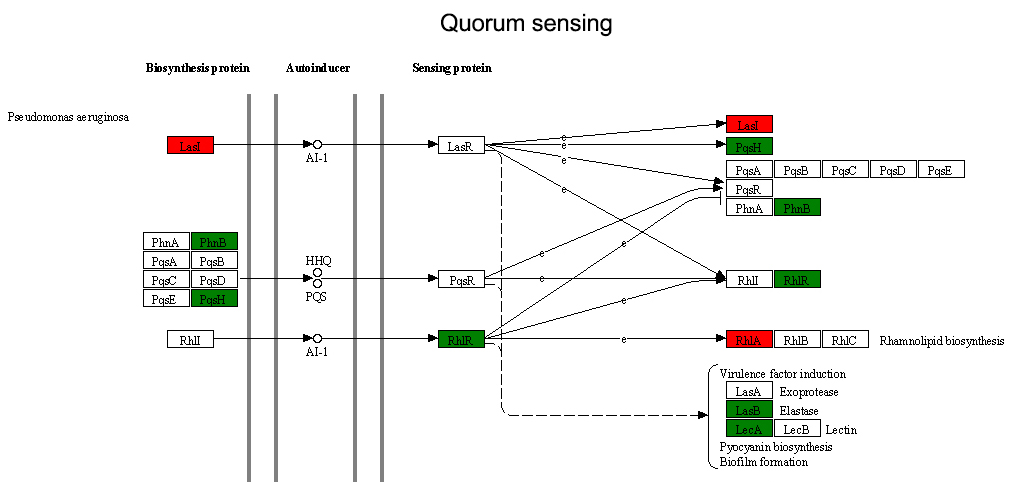
**

**Fig. S8**


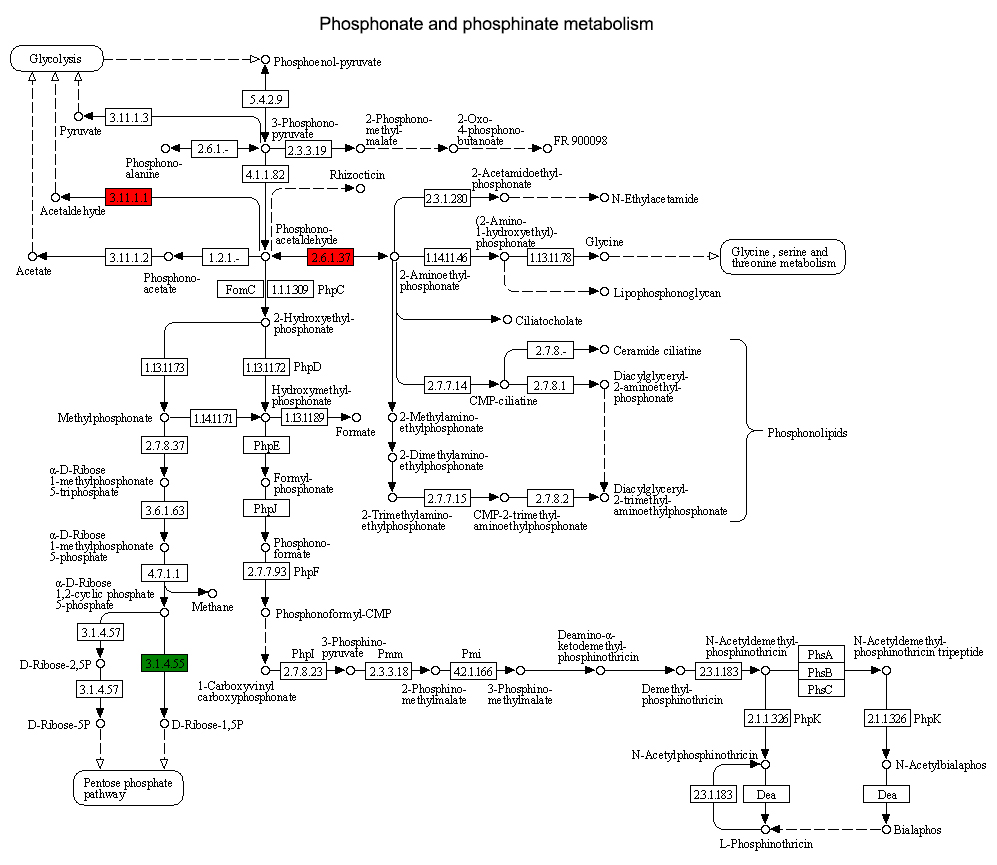


**Fig. S9**

**
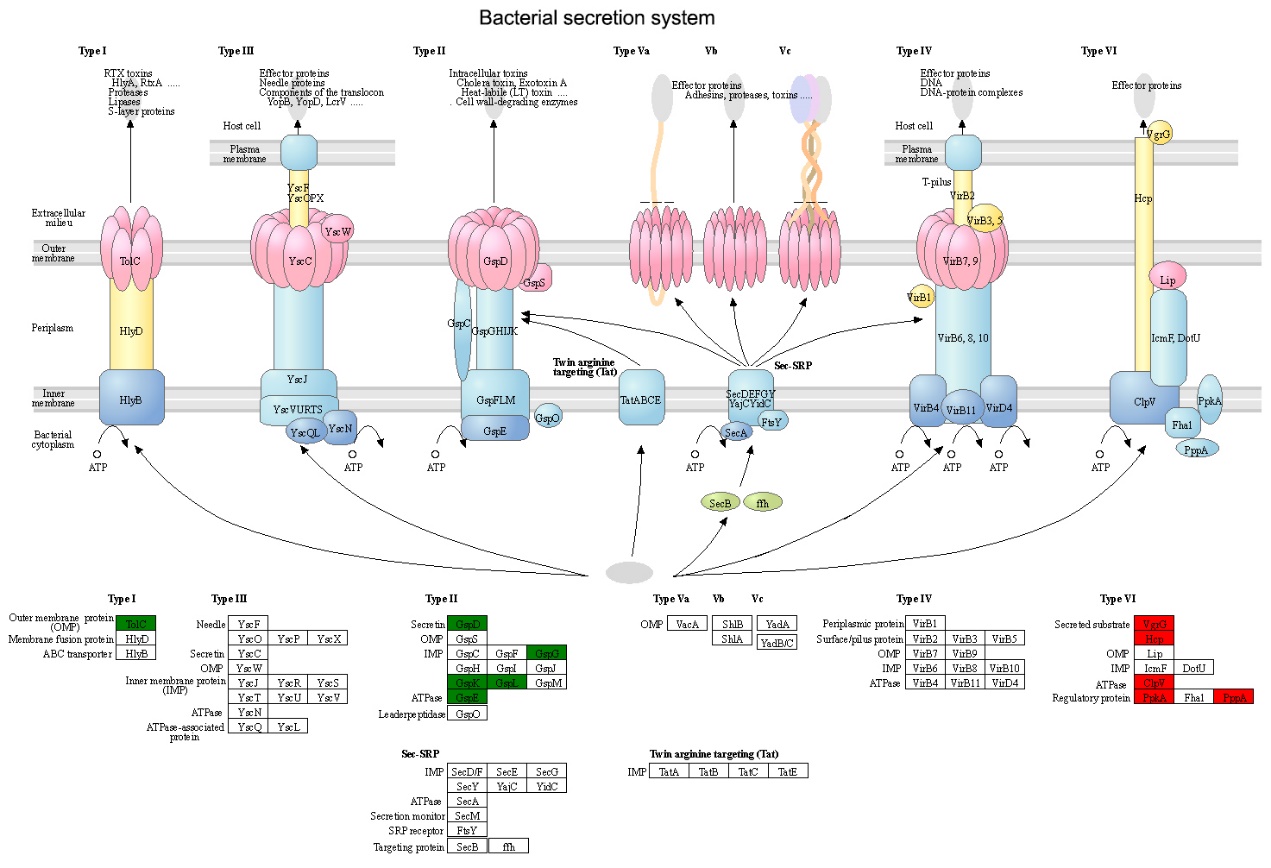
**

**Fig. S10**

**Supplementary Tables**

**Table S1. The peptides and proteins identified by LC-MS/MS**

| Total spectrums | Matched spectrums | Peptides | Unique peptides | Identified proteins | Quantifiable proteins |
| --- | --- | --- | --- | --- | --- |
| 316970 | 57607 | 24063 | 23798 | 3525 | 3115 |

**Table S2.** Identification of differentially expressed proteins in ∆*stk1* strain compared with those in the PAO1 strain by LC-MS/MS.

**Table S3**. GO functional classification of differentially expressed proteins.

**Table S4**. Classification of subcellular localizations of differentially expressed proteins.

**Table S5**. GO enrichment analysis of differentially expressed proteins.

**Table S6**. KEGG pathway enrichment analysis of differentially expressed proteins.

**Table S7**. Protein domain enrichment analysis of differentially expressed proteins.

(Note: Supplemental Table S2-S7 were showed in Excel files (.xlsx))
